# Perivascular spaces are associated with T cell trafficking and may have prognostic relevance in brain metastases

**DOI:** 10.1038/s43856-026-01918-7

**Published:** 2026-10-19

**Authors:** Claudia Maletzki, Eva Michaelis, Sae-Yeon Won, Artem Refaelian, Thomas Freitag, Thomas M. Freiman, Julia Onken, David Wasilewski, Peter Vajkoczy, Jawed Nawabi, Nico Noah Martin, Annette Zimpfer, Daniel Cantré, Florian Gessler, Daniel Dubinski

**Affiliations:** 1 Department of Internal Medicine — Clinic and Polyclinic for Hematology, Hemostaseology, Oncology, Stem Cell Therapy and Palliative Medicine, Rostock University Medical Center, Rostock, Germany; 2 Department of Neurosurgery, Rostock University Medical Center, Rostock, Germany; 3 Department of Neurosurgery, Campus Mitte, Charité-Universitätsmedizin Berlin, Humboldt-Universität zu Berlin, Freie Universität Berlin, Berlin Institute of Health, Berlin, Germany; 4 Department of Neuroradiology, Charité-Universitätsmedizin Berlin, Humboldt-Universität zu Berlin, Freie Universität Berlin, Berlin Institute of Health, Berlin, Germany; 5 Institute of Pathology, Rostock University Medical Center, Rostock, Germany; 6 Department of General, Visceral, Thoracic, Vascular and Transplantation Surgery, Rostock University Medical Center, Rostock, Germany

**Keywords:** Tumour biomarkers, Neurology

## Abstract

**Background:**

Perivascular structures, in particular, perivascular spaces (PVS) are emerging key immune modulators in the tumor microenvironment. However, their relationship with immune infiltration, vascular remodeling, and clinical outcomes in brain metastases is unclear.

**Methods:**

In this retrospective two-center study, we analyzed 56 brain metastases. Perivascular spaces were graded on preoperatively T2-weighted MRI in the basal ganglia and centrum semiovale. Tumor immune infiltration, vascular features, and immune cell phenotypes were assessed by immunohistochemistry and spectral flow cytometry and correlated with clinical outcome.

**Results:**

Here we show that high PVS burden in the centrum semiovale region, but not in the basal ganglia, were significantly associated with improved overall survival. High PVS are linked to increased presence of tumor-infiltrating cytotoxic T cells—most of which exhibited an activated/exhausted phenotype, indicated by PD-1- and Fas-positivity—was likewise prognostically favorable. Moreover, these cells showed elevated expression of the migration marker CXCR3, as well as Granzyme B/IFNγ consistent with effector polarization. High PVS were additionally associated with a higher M1/M2-like macrophage ratio, which showed similar prognostic relevance as high vascular density, suggesting enhanced blood supply and immune cell recruitment.

**Conclusion:**

These findings could establish PVS as a non-invasive imaging marker of anti-tumor immunity and prognosis in patients with brain metastasis, underscoring their potential as biomarkers for immune activity and therapeutic monitoring.

## Plain language summary

Brain metastases are cancer deposits in the brain and are often difficult to treat. In this study, we asked whether small fluid-filled spaces around brain blood vessels, called perivascular spaces, are linked to immune activity in these tumors and to patient survival. We studied brain scans and tumor samples from 56 patients treated at two hospitals. We found that patients with more visible perivascular spaces in one brain region often had more tumor-infiltrating immune cells and lived longer. These immune cells showed signs of an active anti-tumor response. Our findings suggest that perivascular spaces seen on routine brain scans may help identify patients whose tumors show stronger immune activity. Larger studies are needed to confirm this.

## Introduction

Brain metastases (BM) represent the most common intracranial malignancies in adults, occurring in up to 30% of patients with systemic cancer, particularly those with lung, breast, and melanoma primaries. Despite advances in local and systemic therapies, the prognosis for patients with brain metastases remains poor, with median overall survival often limited to a few months. A growing body of evidence indicates that the central nervous system (CNS) is not immunologically inert, but rather a dynamic compartment with complex tumor-immune interactions. Understanding the cellular and spatial architecture of the brain metastatic microenvironment is essential to develop effective therapeutic strategies.

Unlike primary brain tumors, including glioblastoma, BMs must pass through the blood–brain barrier, adapt to the neural milieu, and evade CNS immune surveillance. During this process, metastatic tumor cells co-opt local vessels and recruit stromal elements—including astrocytes, microglia, perivascular macrophages, and endothelial cells—forming a supportive niche that facilitates colonization and progression^1^. Perivascular spaces (PVS) are increasingly recognized as integral components of this metastatic niche.

PVS^2^, also known as Virchow–Robin spaces, are fluid-filled compartments surrounding arterial vessels crossing the deep gray nuclei and white matter of the brain, and participate in interstitial fluid management and immune surveillance^3–6^. They have been identified as dynamic immune niches where macrophages, T cells, and endothelial cells communicate. Under pathological conditions, PVS appear hyperintense on MRI due to their linear structure and fluid-like properties^5^. PVS are commonly associated with aging, hypertension, and neuroinflammation—factors linked to cognitive decline and fatigue^7,8^, but their prevalence, functional role, and prognostic significance in BM remain to be systematically investigated. Preliminary evidence suggests PVS could be tied to tumor-induced compression, glymphatic dysfunction, and impaired fluid dynamics, potentially serving as biomarkers for disease progression or therapeutic response^9,10^.

Recent advances in spatial profiling, multi-parameter flow cytometry, and whole-slide imaging have enabled a more detailed interrogation of the brain metastatic microenvironment, guiding the identification of biomarkers for tailoring precision oncology^11–14^. Besides, these technologies offer the opportunity to correlate radiographic findings such as PVS with immune cell composition, vascular density, and clinical outcomes. In this study, we integrate multimodal imaging, histopathology, and immune phenotyping to investigate the functional relevance of PVS in patients with BM. We show that high PVS burden in the centrum semiovale is associated with improved overall survival and an inflamed tumor microenvironment characterized by cytotoxic T-cell infiltration, checkpoint expression, an increased M1/M2-like macrophage ratio, and endothelial activation. These findings support PVS as non-invasive imaging markers of anti-tumor immune activity and prognosis in patients with brain metastases.

## Methods

### Patient material

Patients were recruited at two university hospitals. The study was conducted in accordance with the ethical standards of the institutional research committees of the university medical centers and with the 1964 Declaration of Helsinki and its later amendments or comparable ethical standards. Ethical approval was obtained from the Ethics Committee of Charité University Medical Center Berlin (approval no. EA1/399/20) and the Ethics Committee of Rostock University Medical Center (approval no. A-2018-0167). Written informed consent was obtained from all participants prior to their inclusion in the study. Clinical, pathological, and anamnestic data were systematically recorded (data cutoff: July 31st, 2025). Tumor material was obtained fresh from surgery and either fixed in 4% formalin or stored vitally (in freezing medium: FCS + 10% DMSO) in liquid nitrogen as described before^15^. To ensure consistency and minimize sampling-related bias, tissue collection was restricted to representative viable tumor regions identified intraoperatively, with avoidance of necrotic or non-tumor tissue whenever feasible.

### PVS measurement from MRI images

PVS measurement was conducted as previously described^2^ by EM and AR, who were blinded to participants’ demographic and clinical information. Briefly, scans were evaluated using T2-weighted axial or coronal MRI sequences. PVS were rated in two distinct anatomic regions: the basal ganglia (BG) along the course of lenticulostriate arteries and the centrum semiovale (CSO) underneath the cerebral cortex, running centripetally from the external aspect of the white matter towards the lateral ventricles. Severity was determined based on individual PVS counts, with each region graded separately as follows: grade I (<10 PVS), grade II (10–20), grade III (20–40), and grade IV (>40)^16,17^. For further stratification, PVS were dichotomized into PVS-high and PVS-low.

### Immunohistochemistry

All samples were studied by an expert pathologist. Histopathology of formalin-fixed paraffin-embedded tumors followed standard protocols, including HE staining and immunohistochemistry. Therefore, 2 µm slides were prepared and samples were stained with a panel of antibodies targeting immune-related surface molecules: anti-CD8 (Clone C8/144B, Dako, Hamburg, Germany, dilution 1:100), anti-CD4 (4B12, dilution 1:100), anti-PD-L1 (Clone 22C3, Dako, dilution 1:100), anti-CD34, anti-FOXP3, anti-CD68, anti-CD163 (all from Dako, dilution/antibody 1:100). Immune cell quantification was done using QuPath. For this, the machine-learning tool Pixel Classifier was applied, which is commonly used for membrane staining analysis. The classifier was trained using a supervised learning approach (Random Forests), enabling each pixel to be assigned to a user-defined class.

### CD45 enrichment of tumor-infiltrating leukocytes

Tumor-infiltrating immune cells were isolated from vitally stored tumor tissue using CD45 MicroBeads (Miltenyi Biotec, Cat: 130-118-780) according to the manufacturer’s protocol. Briefly, tumor specimens were carefully thawed in a 37 °C water bath for 2–3 min, transferred to a Petri dish, and the cryovial was rinsed with 1 mL warm 1× PBS, added dropwise (1 drop every 5 s) to minimize osmotic lysis. The tissue was then gradually diluted with PBS in a 1:1 ratio, allowing ~1 min between additions. The tissue was homogenized using a 100 µm cell strainer placed on a 50 mL polypropylene tube using a 10 mL syringe plunger, followed by 2–3 rinses with 1x PBS. The filtrate was passed through a 40 µm cell strainer and rinsed again 1–2 times. Cells were pelleted by centrifugation (5 min, 350 *× g*), resuspended in DNase solution, and incubated for 45 minutes at room temperature. A second centrifugation (5 min, 350 *× g*, 4 °C) was performed, and the pellet was washed in 200 µL wash buffer (2% BSA + 2 mM EDTA in 1× PBS; 5 min, 500 *× g*) before decanting the supernatant. Cells were incubated with anti-CD45 MicroBeads for 15 min at 4 °C. After labeling, the cell suspension was loaded onto an MS magnetic separation column placed in a MACS Separator (Miltenyi Biotec, Bergisch Gladbach, Germany). CD45⁺ cells were retained in the column and subsequently eluted following removal from the magnetic field. Downstream analysis was performed on the enriched CD45⁺ cell fraction, and, in some experiments, the CD45⁻ cell fraction.

### Spectral flow cytometry

Viability staining on CD45^+^ and CD45^-^ cell fractions was done first by resuspending cell pellets in 100 µL of Fixable Viability Dye eFluor™ 450 (Thermo Fisher; 1:2000 1x PBS) or Zombie NIR^TM^ (Biolegend, 1:5000 in 1x PBS). The samples were incubated for 30 min at room temperature in the dark, centrifuged (5 min, 500 *× g*), washed with 200 µL wash buffer, and centrifuged again under the same conditions. For blocking, samples were incubated for 10 min at room temperature in the dark with a monocyte blocker mix (87.5 µL PBS, 10 µL FBS, and 2.5 µL True-Stain Monocyte Blocker™ (Biolegend, San Diego, USA) per sample). Extracellular staining was performed by adding 50 µL BD Horizon™ Brilliant Stain Buffer (BD Biosciences, Heidelberg, Germany) to each sample, followed by addition of a pre-centrifuged (5 min, 10,000 *× g*) antibody master mix, with the following monoclonal antibodies (mAbs) used for CD45^+^ cell fractions: Panel 1: anti-CD279 (PD1) BV421 (clone: NAT105, Biolegend, dilution 1:500), anti-CD45R0 BV480 (clone: UCHL1, BD Bioscience, dilution 1:62.5), anti-CD14 BV510 (clone: 63D3, Biolegend, dilution 1:125), anti-CD8 BV570 (clone: RPA-T8, Biolegend dilution 1:62.5), anti CD19 BV605 (clone: HIB19, Biolegend, dilution 1:125), anti-CD16 BV650 (clone: 3G8, Biolegend, dilution 1:125), anti-CD56 BV711 (clone: 5.1H11, Biolegend, dilution 1:300), anti-CD4 BV750 (clone: Sk3, Biolegend, dilution 1:625), anti-CD11c BV785 (clone: 3.9, Biolegend), anti-CD3 Spark Blue 550 (clone: SK7, Biolegend, dilution 1:100), anti-CD25 PE (clone: REA570, Miltenyi Biotec, dilution 1:200), anti-CD95 PE/Cy5 (clone: DX2, Biolegend, dilution 1:625), anti-CD163 PerCP/Cy5.5 (clone: RM3/1, Biolegend, dilution 1:625), anti-CD177 PE-Vio770 (clone: REA258, Miltenyi Biotec, dilution 1:200), anti-CD44 PE/Fire™ 810 (clone: IM7, Biolegend, dilution 1:300), anti-CD206 APC (clone: 15-2, Biolegend, dilution 1:200), anti-CD20 Spark NIR 685 (clone: 2H7, Biolegend, dilution 1:625), anti-CD127 APC-R700 (clone: HIL-7R-M21, BD Biosciences, dilution 1:200), anti-CD45RA APC/Fire750 (clone: HI100, Biolegend, dilution 1:625), anti-CD11b APC/Fire810 (clone: M1/70, Biolegend, dilution 1:625). Panel 2 included the following mAbs: anti-CD45 BV510 (clone:2D1, Biolegend; dilution 1:50), anti-CD3 Spark Blue 550 (clone: SK7, Biolegend), anti-CD4 BV750 (clone: Sk3, Biolegend, dilution 1:100), anti-CD8 BV570 (clone: RPA-T8, Biolegend, dilution 1:100), anti-CD279 BV421(clone: NAT105, Biolegend, dilution 1:100), anti-TIM-3 BUV737 (clone: 7D3, Biolegend, dilution 1:100), anti-CD183 (CXCR3) RB780 (clone: 1C6, BD Biosciences, dilution 1:100). Additionally, CD45^−^ cell fractions were stained using the following mAbs in Panel 3: anti-CD45 APC (clone: HI30, Cytek Biosciences, dilution 1:100), anti-CD309 (VEGFR-2) BV421 (clone: A16085H, Biolegend, dilution 1:100), anti-CD31 (PECAM-1) cFluor V475 (clone: WM59, Cytek Biosciences, dilution 1:100), anti-CD106 (VCAM-1) BV605 (clone: 51-10C9, BD biosciences, dilution 1:100), and anti-CD54 (ICAM-1) PE (clone: 15,2, Cytek Biosciences, dilution 1:100). Samples were vortexed, incubated for 30 min in the dark at room temperature, washed twice with 0.5 mL wash buffer (centrifugation: 5 min, 500 *× g*) and resuspended in 300 µL PBS for storage at 4 °C until flow cytometric analysis. For intracellular staining of CD45^+^ and CD45^−^ cell fractions (Panel 2 and Panel 3), Foxp3/Transcription Factor Staining Buffer Set (eBioscience™) was used according to the manufacturer’s instructions. The following mAbs were used thereafter: anti-Granzyme B APC-Cy7 (clone: QA16A02, Biolegend, dilution 1:100), anti-IFN-γ Alexa Fluor 647 (clone: 4S.B3, Biolegend, dilution 1:100) for the CD45^+^ cell fraction and anti-CXCL9 Vio B515 (clone: REA1300, Miltenyi Biotec, dilution 1:100), and anti-CXCL10 PerCP-eFluor 710 (clone: 4NY8UN, ThermoFisher Scientific, dilution 1:100). Staining was done overnight at 4 °C, followed by two washing steps and resuspension of the cell pellet in washing buffer. Measurement was conducted on a spectral flow cytometer (3L-Cytek™ Aurora). Data were analyzed using SpectroFlow™ Version 3.3 and FlowJo™ Version 10.6.1.

### Statistics and reproducibility

All statistical analyses were performed using GraphPad Prism (version 10.4.1, GraphPad Software, San Diego, CA), unless otherwise specified. Data were tested for normality using the Shapiro–Wilk test. For normally distributed data, differences between two groups were assessed using unpaired two-tailed Student’s t-tests. For non-normally distributed data, the non-parametric Mann–Whitney U test was applied. For parameters identified as significantly different in flow cytometry, p-values from multiple pairwise comparisons were adjusted for multiple testing using the two-step linear step-up procedure of Benjamini, Krieger, and Yekutieli false discovery rate (FDR) procedure. The FDR was controlled across all simultaneous tests within each analysis family. Adjusted *q*-values < 0.05 were considered statistically significant.

Kaplan–Meier survival curves were generated to evaluate differences in overall survival between groups, and statistical significance was determined using the log-rank (Mantel–Cox) test. Data are presented as mean ± standard deviation (SD), unless otherwise noted. *P*-values < 0.05 were considered statistically significant.

Sample sizes are indicated in the corresponding Methods sections and figure legends. Immunohistochemical and flow cytometric analyses were performed on available patient-derived tumor samples, with each patient sample considered an independent biological replicate. No technical replicate measurements were pooled as independent observations.

### Ethics

The study was conducted in accordance with the ethical standards of the institutional research committees of the Charité Berlin and the Rostock University Medical Center, and with the 1964 Declaration of Helsinki and its later amendments or comparable ethical standards. Ethical approval was obtained from the ethics committees of both institutions ([Berlin Ethics Committee Approval No: EA1/399/20], [Rostock Ethics Committee Approval No: A-2018-0167]). Written informed consent was obtained from all participants prior to their inclusion in the study.

## Results

### Patient characteristics of the cohort

**Fig. 1:**
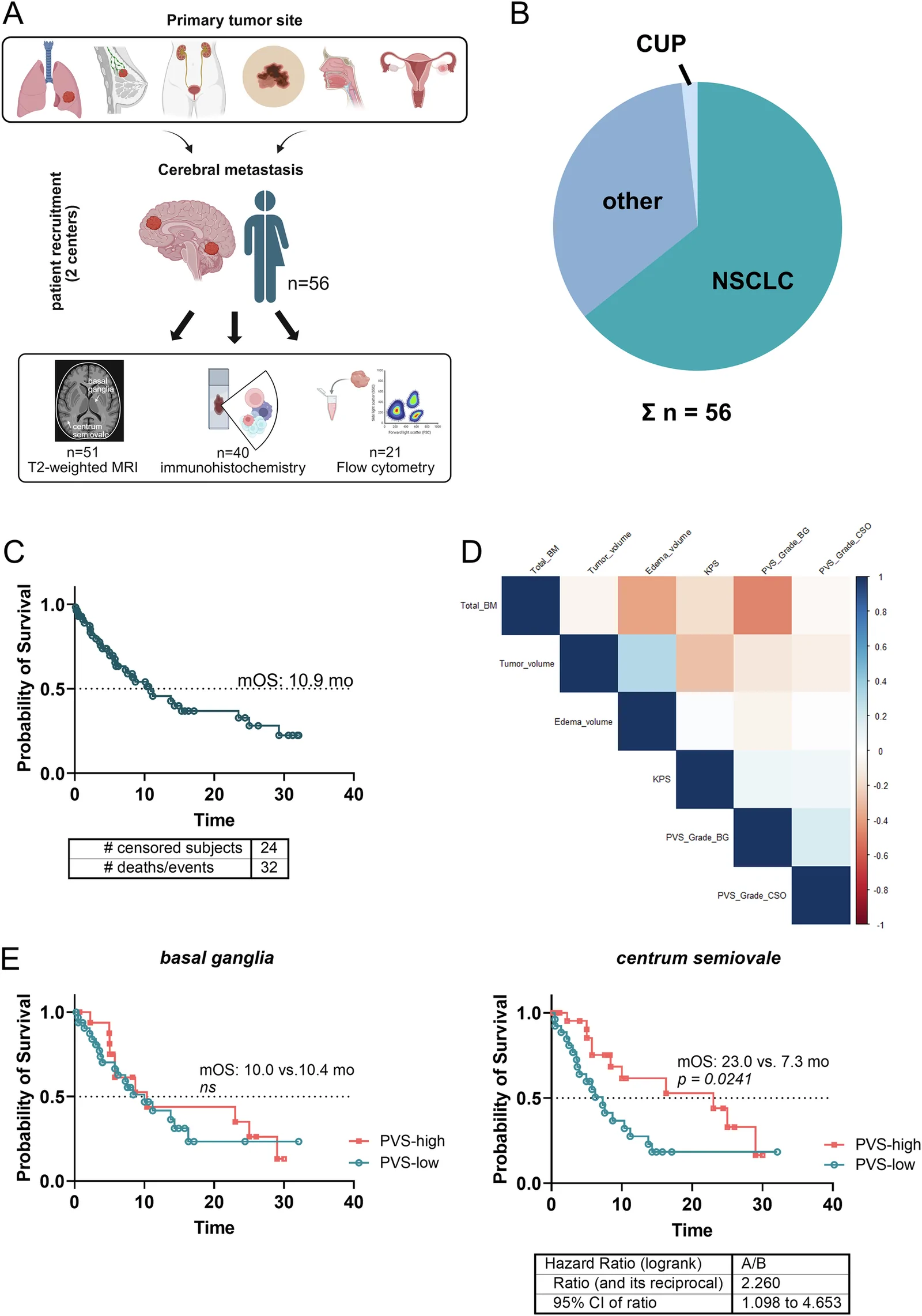
Study overview and outcome of patients with brain metastases (BM). **A** Workflow and patient cohort (created with BioRender). **B** Distribution of primary extracranial anatomic sites. **C**, **E** Kaplan Meier survival curves. **C** Overall survival of patients with BMs, independent of primary extracranial tumor location. *N* = 56. **D** Spearman correlation analysis stratified by age and PVS grade. *N* = 51. **E** Overall survival stratified by PVS grade. *N* = 51. PVS were rated in two distinct anatomic regions: the basal ganglia (BG) and the centrum semiovale (CSO). PVS were dichotomized into PVS-high and PVS-low. **D** Spearman correlation analysis showing the association between PVS grade and clinicopathological characteristics. Data were available from n = 26 patients. **E** Log rank analysis; *p* < 0.05 vs. PVS-low. mOS median overall survival, PVS perivascular spaces. Data cutoff: July 31st, 2025.

Baseline demographic characteristics are summarized in Table 1. A total of 56 patients were included (Fig. 1A). The median age at diagnosis was 67.8 years (±11.2 years), with 60.7% male and 39.3% female. The mean Graded Prognostic Assessment (GPA) score, available for 30 patients, was 2.3 (±0.8), indicating intermediate prognosis. The majority had primary non-small cell lung cancer (NSCLC, 64.3%; Fig. 1B), but other extracranial primary tumor sites were also present. Extracranial tumor resection prior to BM surgery was performed in 20 patients. BM were most frequently located in the cerebellum or frontal lobe, with five patients presenting with multifocal disease. At the time of inclusion, 44 patients were treatment-naïve with respect to systemic therapy or radiation, while 12 had received prior treatment. Most patients subsequently received adjuvant therapy, including chemotherapy, targeted agents, immune checkpoint inhibitors, or radiotherapy (Table 1). The median overall survival following diagnosis was 10.9 months (Fig. 1C).

**Table 1:** Demographic and clinical characteristics of the patient cohort

Baseline Clinical Characteristics	Total (n=56)
Age, median (range)	67.8 (±11.2)
Sex, *n* (%)	
Female	22 (39.3)
Male	34 (60.7)
GPA, mean (range)	2.3 (0.8)
Primary tumor, number (%)	
Bronchial-Ca	
Non-small cell lung-Ca	36 (64.3)
Small cell lung-Ca	2 (3.6)
Urogenital-Ca	6 (10.7)
Breast-Ca	2 (3.6)
Malignant Melanoma	2 (3.6)
Others^a^	7 (12.5)
Unknown	1 (1.7)
Anatomic site, number (%)	
Cerebellar	12 (22.6)
Frontal	11 (20.8)
Infratentorial	1 (1.9)
Temporal	5 (9.4)
Occipital	7 (13.2)
Parietal	9 (17.0)
Parieto-occipital	3 (5.7)
Multifocal	5 (9.4)
Glucocorticoid usage, number (%)	
Yes	29 (51.8)
No	27 (48.2)
Resection of primary tumor before metastasis resection, number (%)	
Yes	20 (35.7)
No	31 (55.4)
unknown	2 (3.6)
Resection of primary tumor after 1st metastasis resection, number (%)	3 (5.3)
Treatment-naïve at time of first resection^b^, number (%)	
Yes	44 (78.6)
No	12 (21.4)
Adjuvant therapy, number (%)	
Ctx or targeted therapy	13 (23.2)
Immunotherapy^c^	14 (25.0)
Combined chemo-immunotherapy	4 (7.1)
Radiotherapy^d^	44 (78.6)
PVS grading, number (%)	
*Basal ganglia*	
Grade I/II	35 (67.3)
Grade III/IV	17 (32.7)
*Centrum semiovale*	
Grade I/II	28 (53.8)
Grade III/IV	24 (46.2)

*Ca* cancer, *Ctx* chemotherapy (not specified, depending on primary cancer location).

^a^Including squamous cell-Ca (oral cavity), ovarial-Ca, endometrial-Ca, esophagus-Ca, and CUP.

^b^Naive for systemic pharmacologic pre-treatment or radiation.

^c^Immune-checkpoint-inhibitor (anti-PD1, anti-PD-L1, anti-CTLA-4).

^d^Includes conventional radiation, whole brain radiation, intraoperative radiation, and stereotactic radiosurgery.

### PVS are associated with favorable outcome

PVS grading was performed on T2-weighted MRI acquired prior to surgery. MRI images were available for 55 patients; in one case, no MRI was performed, and in four cases, the data were not evaluable. Consequently, data from 51 patients were included in the analysis. PVS in the BG and CSO were assessed and dichotomized for statistical analysis into PVS-low (grades I–II) and PVS-high (grades III–IV, representative images are shown in supplementary Fig. 1). Initial analysis showed no correlation between PVS grade, and clinicopathological characteristics, including Karnofsky performance status, total number of BM, tumor/edema volume and age, ruling out these parameters as a potential confounding factors (Fig. 1D).

PVS (defined as PVS-high) was detected in 32.7% of patients in the BG and 46.2% in the CSO (Table 1). In the BG, PVS were not prognostic, with outcomes comparable between PVS-high and PVS-low groups (Fig. 1E left). In contrast, patients with PVS-high in the CSO exhibited significantly improved outcomes compared to those without enlargement (*p* < *0.05*, Fig. 1E right). The median overall survival was 23.0 months in the PVS-high group versus 7.3 months in the PVS-low group. Given that the GPA score is a potential confounder of prognosis, the analysis was repeated with stratification by GPA. Data were available for 26 patients, of whom 25 had primary NSCLC and one had SCLC. Despite the limited sample size, PVS remained a significant prognostic factor, with patients exhibiting high PVS in the CSO region showing significantly prolonged survival compared to those with low PVS (Supplementary Fig. 2). Then, a Cox proportional hazards regression model was fitted to these 26 patients, of whom 17 (65.4%) experienced the event and 9 (34.6%) were censored (Table 2). In the adjusted analysis, both GPA and PVS in the CSO region emerged as independent predictors of overall survival. Higher GPA was significantly associated with a reduced hazard of death (hazard ratio [HR] 0.29, 95% confidence interval [CI] 0.08–0.88). Similarly, higher PVS scoring CSO was associated with improved survival (HR = 0.44; 95% CI: 0.19–0.92). In contrast, the other factors were not significantly associated with the outcome. Given the limited sample size and number of events, the precision of several estimates was constrained, particularly for non-significant variables. Nonetheless, the consistent and statistically significant associations observed for GPA and, notably, the new prognosticator PVS in the CSO region support their role as prognostic factors for overall survival in this cohort.

**Table 2:** COX regression analysis on relevant clinical parameters to identify prognostic factors for overall survival in this cohort

Parameter estimates	Variable	Estimate	95% CI (profile likelihood)
β1	underlying other diseases	0.3508	−1.013 to 1.889
β2	age	0.03443	−0.02035 to 0.09144
β3	GPA	−0.2243	−0.9822 to 0.5268
β4	PVS Scoring CSO	−0.8237	−1.674 to −0.07919
β5	PVS Scoring BG	0.2215	−0.5791 to 1.003

Hazard ratios	Variable	Estimate	95% CI (profile likelihood)
exp(β1)	underlying other diseases	1.420	0.3632 to 6.610
exp(β2)	age	1.035	0.9799 to 1.096
exp(β3)	GPA	0.799	0.3745 to 1.693
exp(β4)	PVS Scoring CSO	0.438	0.1875 to 0.9239
exp(β5)	PVS Scoring BG	1.248	0.5604 to 2.726

Data summary	
Rows in the table	26
Rows skipped (missing data)	0
Rows analyzed (#observations)	26
Number of ties	0
Number of censored	9
Number of deaths/events	17
Ratio	0.529
Number of censored	9
Number of observations	26
Ratio	0.340
Number of deaths/events	17
Number of parameter estimates	5
Ratio	3.400

*GPA* Graded Prognostic Assessment.

### The tumor-infiltrating immune landscape correlates with patients’ prognosis

To investigate whether improved patient outcomes were associated with tumor infiltration, detailed immune profiling was performed. Depending on sample preservation, either immunohistochemistry (for paraffin-embedded tissue, mostly NSCLC-derived metastases, n = 40) or flow cytometry (for frozen material, NSCLC and other tumors, n = 21) was conducted. In some cases (n = 7), both methods could be applied.

**Fig. 2:**
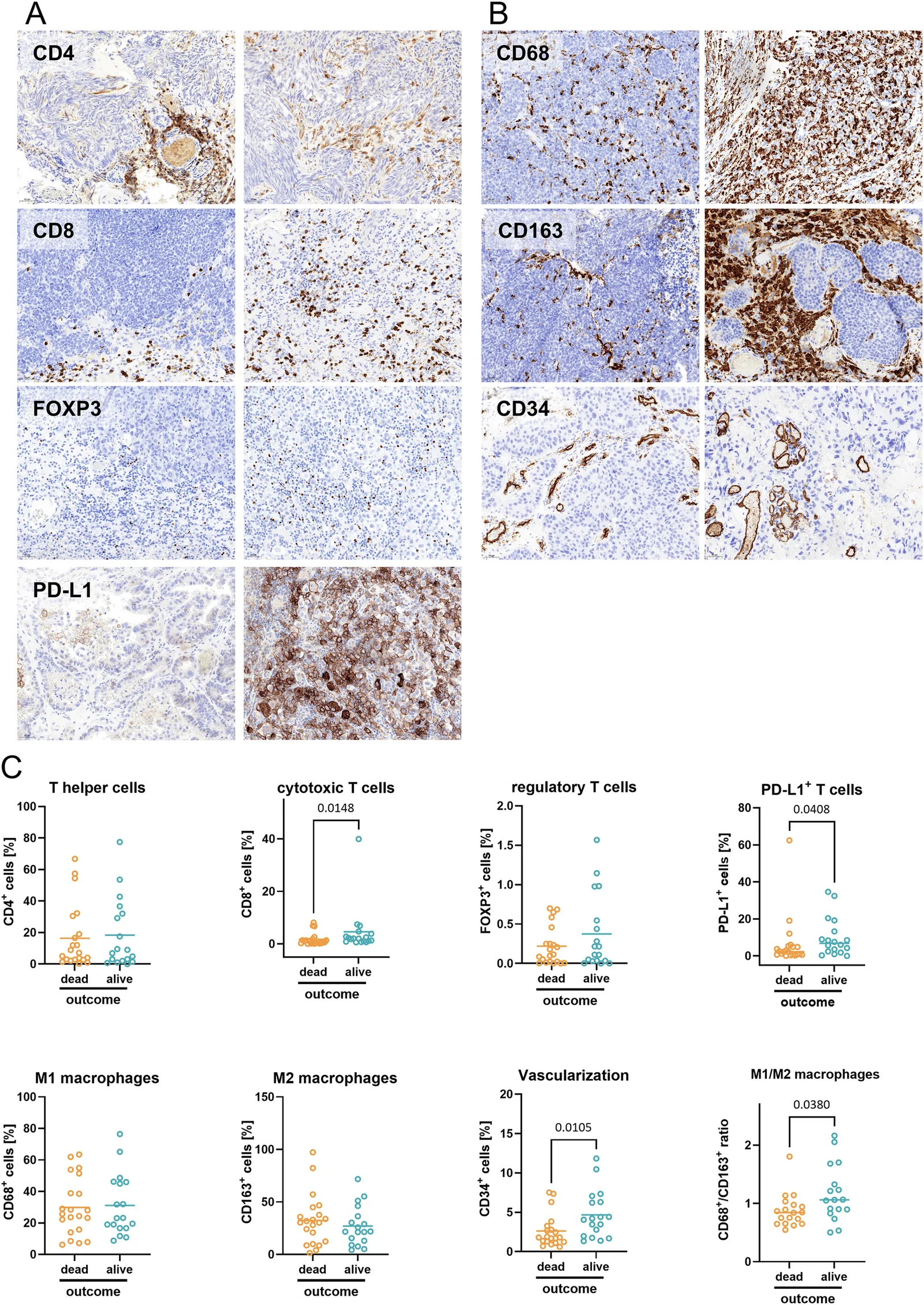
Immunohistochemistry of BM samples. **A**, **B** Representative images of whole tumor slides stained for specific immune cells: T cells (CD4, CD8, FOXP3, PD-L1), myeloid cells (CD68, CD163), and endothelial cells (CD34). Original magnification 10×. Scale bar: 100 µm. **C** Quantification and stratification by patient’s outcome (dead/alive). Quantification of specific cell types was done using QuPath. Given is the % of infiltrating cells/slide. For M1/M2-like macrophages, the ratio was calculated by dividing the value of CD68^+^ M1-like macrophages by the value of CD163^+^ M2-like macrophages. *N* = 40, Unpaired two-tailed Student’s *t* test or Mann–Whitney U test, * *p* < 0.05.

Immunohistochemical analysis was performed on whole tumor slides (Fig. 2A, B, and supplementary Fig. 3). First, immune cell infiltration patterns were assessed across whole-slide images, stratified by either total or intratumoral infiltration. No significant differences were observed using this stratification approach (exemplary data shown in Supplementary Fig. 4). Therefore, subsequent analyses were conducted on whole-slide data, and the total number of infiltrating immune cells per case was quantified.

Using this approach, we observed substantial inter-individual variability in T cell infiltration. Across cases, CD4⁺ T helper cells were more abundant than CD8⁺ cytotoxic T cells (Fig. 2C). Regulatory T cells (Tregs), identified by FOXP3 expression, were present in all tumors, but their infiltration was low (<1%), and the distribution was irregular (Fig. 2A). Most tumors showed PD-L1-positivity on tumor and/or immune cells and all tumors exhibited pronounced monocyte infiltration (CD68^+^, CD163^+^), which was either focal or diffuse. In certain cases, monocytes formed dense clusters surrounding tumor cells (Fig. 2B), suggesting a significant role in the tumor microenvironment. Also, tumor vascularization, indicated by CD34 positivity, a specific marker for endothelial progenitors, differed between individual cases (Fig. 2B).

Analysis of the immune infiltration patterns in relation to patient outcomes revealed a significant prognostic association with the presence of CD8^+^ T cells and PD-L1 (*p* < 0.05 for deceased patients *vs*. survivors, Fig. 2C). Both markers were found to be significantly more abundant in survivors. Additionally, the M1/M2-like macrophage ratio demonstrated a clear prognostic value, with a higher ratio correlating with improved outcome (*p* < 0.01 for deceased *vs*. surviving patients). Tumor vascularization also showed a similar positive prognostic association (*p* < 0.05, deceased *vs*. alive).

**Fig. 3:**
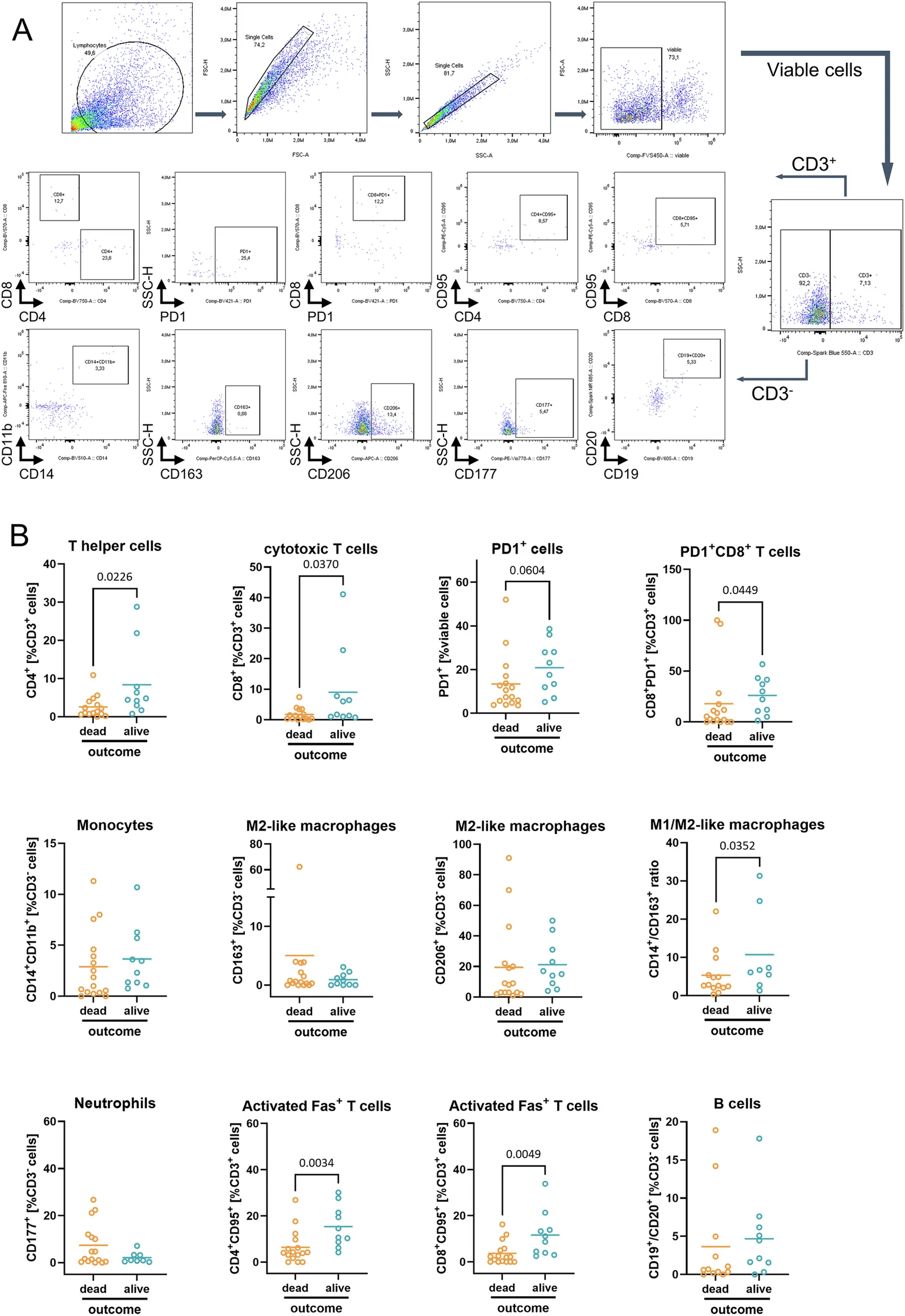
Flow cytometry of BM samples. Tumor-infiltrating immune cells were isolated from vitally stored tumor tissue using CD45 MicroBeads as described in “Materials and Methods”. Spectral flow cytometry was done following staining against a panel of surface antigens to discriminate immune cell subtypes. **A** Gating strategy. **B** Quantification and stratification by patient’s outcome (dead/alive by data cutoff: July 31st 2025). Specific immune cell subtypes were identified by their expression profile. Given is the % of positive cells after gating of the viable immune cells with additional hierarchical sub-gating to distinguish lymphoid and myeloid compartments. For M1/M2-like macrophages, the ratio was calculated by dividing the value of CD3^-^CD14^+^ macrophages by the value of CD3^-^CD163^+^ M2-like macrophages. *N* = 25, Unpaired two-tailed Student’s *t* test or Mann–Whitney U test, * *p* < 0.05.

Accompanying flow cytometry of tumor samples yielded results consistent with the immunohistochemistry findings (Fig. 3). Due to the lack of spatial information regarding the distribution of individual immune cells, we focused on assessing the mean abundance of immune cell populations, the co-expression of immune-related molecules, and their potential prognostic impact on survival. Figure 3A shows the gating strategy for CD45^+^ enriched leukocytes. In general, increased infiltration of CD4^+^ T helper and cytotoxic CD8^+^ T cells was associated with a favorable prognosis (*p* < 0.05, deceased vs. alive, Fig. 3B). The positive prognostic effect for CD8^+^ T lymphocytes remained significant even when considering PD1-status (*p* < 0.05, deceased vs. alive).

Regarding monocytes, identified by the pan-monocyte markers CD14 and CD11b, and M2 macrophages, characterized by CD163 and CD206 expression, we observed that M2-like macrophage infiltration was also prognostically relevant, particularly when evaluating the M1/M2-like macrophage ratio. A higher ratio was positively correlated with a better outcome. Additional important findings, which were not captured by immunohistochemistry, included the identification of CD177^+^ neutrophils, the detection of CD95 (Fas) on tumor-infiltrating lymphocytes and the presence of CD19^+^/CD20^+^ B cells (Fig. 3B). Interestingly, CD95 expression on both CD4^+^ and CD8^+^ T cells was significantly higher in survivors (*p* < 0.01, deceased *vs*. alive), suggesting active immune engagement in these patients. After FDR correction, significant differences across markers remained between PD-1⁺ and both CD4⁺ and CD8⁺ T cell populations (*q* < 0.001), as well as in Fas^+^ expressing T cells. In contrast, no significant differences were detected between other subpopulations (*q* < 0.01) (Supplementary Table 1).

Collectively, these findings highlight that the prognostic value of infiltrating immune cells is determined by their functional quality, rather than their mere quantity.

### High PVS indicates a prognostically favorable, inflamed tumor microenvironment

We next examined whether PVS were associated with local immune infiltration patterns, thereby exploring a potential link between these two independent prognostic markers. The markers previously identified as prognostically relevant were considered here.

**Fig. 4:**
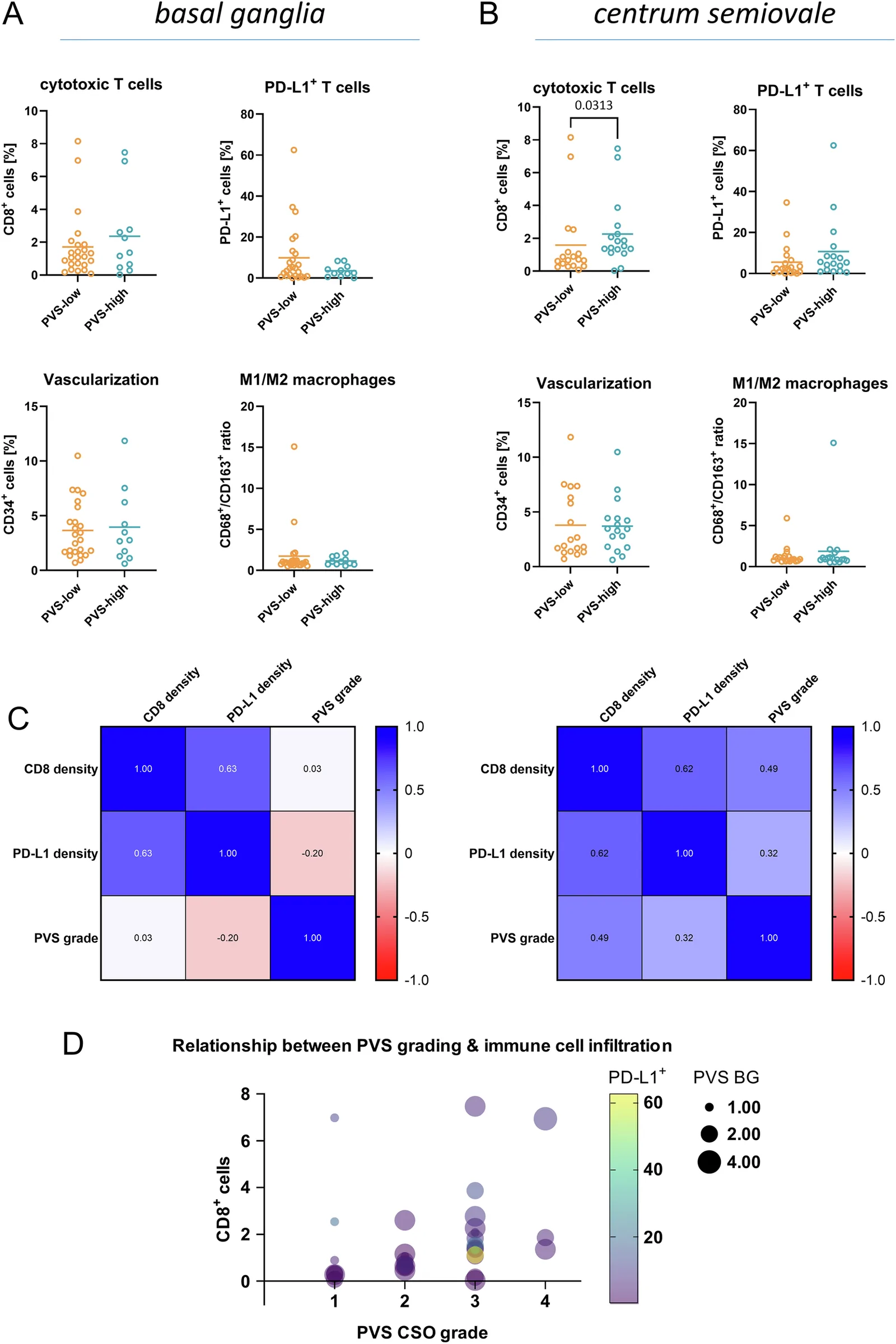
Immunohistochemistry of BM samples stratified by PVS grade. **A** Immune cell infiltration was stratified by MRI-T2 weighted PVS grade in two distinct anatomic locations, i.e. **A** BG and **B** CSO. Therefore, prognostically relevant immune and endothelial cell marker (CD8, PD-L1, CD68, CD163, and CD34), were considered. Immune cell quantification was done using QuPath. Given is the % number of infiltrating cells/slide. For M1/M2-like macrophages, the ratio was calculated by dividing the value of CD68^+^ M1-like macrophages by the value of CD163^+^ M2-like macrophages. *N* = 36, Mann–Whitney U test, * p < 0.05. **C** Spearman correlation analysis stratified by CD8 density, PD-L1 density, and PVS grade. N = 36. **D** Bubble plots showing the association between PVS grade and immune cell infiltration. The bubble size correlates with the PVS grade in the BG region, whereas the color indicates the number of positive cells. *N* = 34.

In the BG, no consistent relationship between PVS and immune infiltration was observed, with staining patterns appearing largely random. Although the PVS-high group tended to show increased numbers of infiltrating cytotoxic T cells and reduced T cell exhaustion indicated by lower PD-L1 expression (Fig. 4A), these differences did not reach statistical significance (Spearman *r* = 0.03 and* −*0.20, respectively, Fig. 4C). Similarly, intratumoral vascularization and the abundance or M1/M2-like macrophage ratio showed no significant correlation with PVS grade. However, BM with high PVS in the CSO region exhibited a non-significant trend toward a more favorable M1/M2-like infiltration profile (Fig. 4B).

In contrast, stratification by PVS grade in the CSO revealed a clear association between PVS grade and tumor immune infiltration (Fig. 4B). PVS-high cases displayed significantly higher numbers of infiltrating CD8^+^ T cells compared with the PVS-low group (*p* < 0.05, Spearman *r* = 0.49, Fig. 4C). Similar associations were observed for PD-L1–positivity (*p* < 0.05, Spearman *r* = 0.32), whereas the intratumoral vascularization did not differ between groups. The M1/M2-like macrophage ratio also tended to increase with PVS grade, though this trend did not reach statistical significance.

**Fig. 5:**
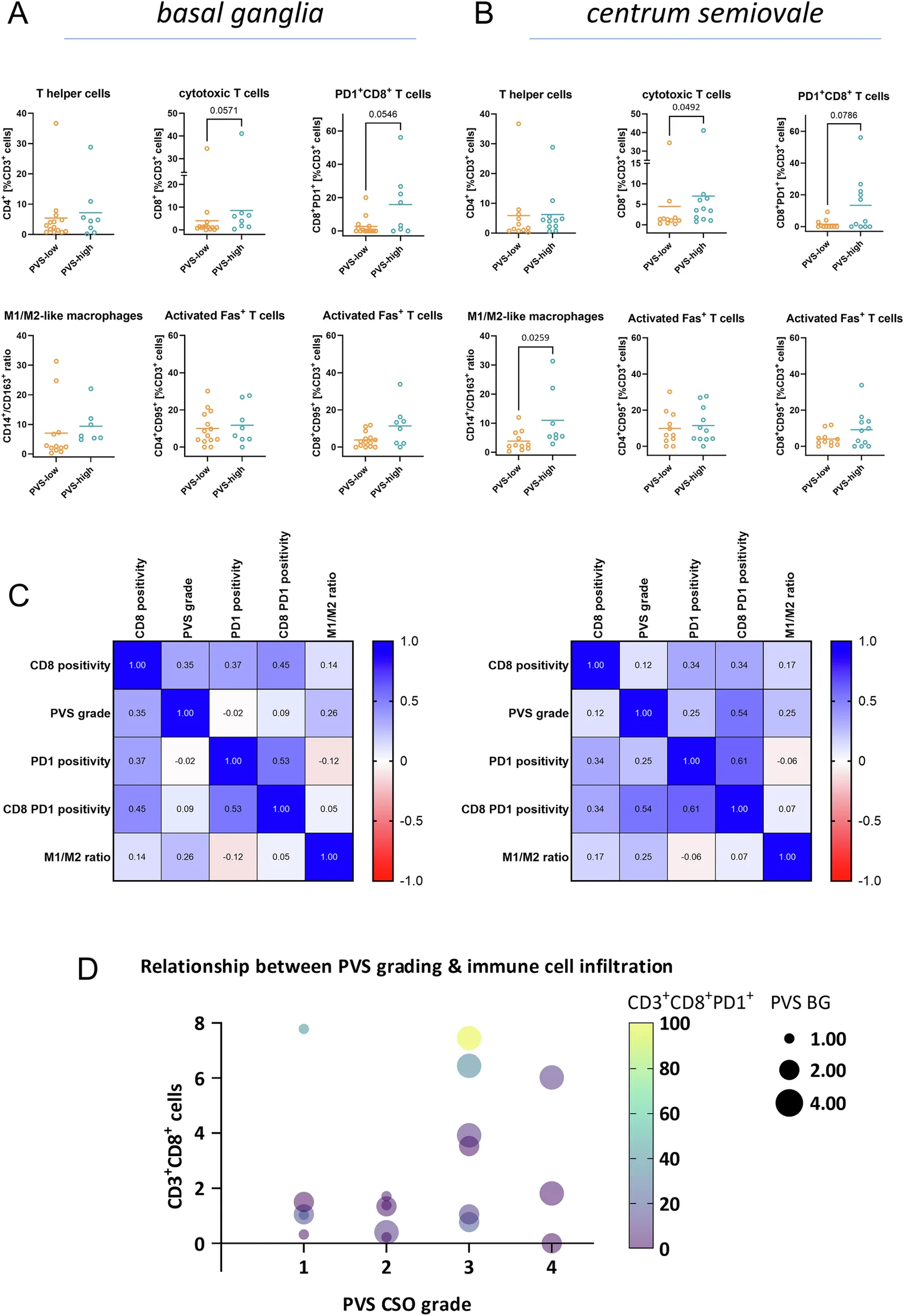
Flow cytometry of BM samples stratified by PVS grade. Immune cell infiltration was stratified by MRI-T2 weighted PVS grade in two distinct anatomic locations, i.e. **A** BG and **B** CSO. Therefore, prognostically relevant immune cell marker (CD4, CD8, PD1, CD14, CD163, and CD95) were considered. Immune cell quantification was done by including the number of viable immune cells, positive for either marker. Given is the % of positive cells after gating of the viable immune cells with additional hierarchical sub-gating to distinguish lymphoid and myeloid compartments. For M1/M2-like macrophages, the ratio was calculated by dividing the value of CD3^-^CD14^+^ macrophages by the value of CD3^-^CD163^+^ M2-like macrophages. *N* = 22, Unpaired two-tailed Student’s *t* test or Mann–Whitney U test, * *p* < 0.05. **C** Spearman correlation analysis stratified by CD8 positivity, PD-L1 positivity, M1/M2-like ratio, and PVS grade. *N* = 18. **D** Bubble plots showing the association between PVS grade and immune cell infiltration. The bubble size correlates with the PVS grade in the BG region, whereas the color indicates the number of positive cells. *N* = 21.

Correlation of PVS grade with flow cytometry data revealed a similar trend in the BG (Fig. 5A). Although not statistically significant, the PVS-high group showed an increased frequency of cytotoxic CD8^+^ T cells, most of which co-expressed PD1 (Spearman *r* = 0.09, Fig. 5C). No other immune markers were associated with PVS grade.

In the CSO, PVS grading further confirmed the link between PVS enlargement and T-cell infiltration (Fig. 5B). Notably, the PVS-high group displayed significantly higher numbers of cytotoxic CD8^+^ T cells compared with the PVS-low group (*p* < 0.05), with the majority of cells co-expressing PD1 (Spearman *r* = 0.54, Fig. 5C). A comparable association was observed for the M1/M2-like macrophage ratio, as higher ratios were accompanied by greater PVS enlargement (*p* < 0.05 vs. PVS-low). By contrast, expression of the activation marker Fas on CD4^+^ and CD8^+^ T cells did not correlate with PVS grade (Fig. 5B). After correction for multiple testing, the analysis of significantly different markers confirmed the robustness of the findings, demonstrating significant differences in PD-1 positivity across PVS grades (*p* < 0.0001) (Supplementary Table 2). Subsequently, the dose–response relationship between PVS grading and immune cell infiltration was evaluated, focusing on the markers identified as significant (i.e., CD8⁺ T cells and PD-L1⁺ cells for IHC, and CD3⁺CD8⁺ T cells and CD3⁺CD8⁺PD1⁺ cells for flow cytometry; Figs. 4D and 5D). Moreover, consistency between the two different methods (i.e. IHC and flow cytometry) was assessed using Pearson’s correlation coefficient (*r*), Spearman’s rank correlation coefficient (*ρ*), and Lin’s concordance correlation coefficient (CCC, Supplementary Fig. 5). By definition, CCC ≤ *r*, and a marked discrepancy between the two (as observed for CD163, *r *= 0.81 vs. CCC = 0.53) indicates good correlation but poor absolute agreement. Given the small sample size, these concordance estimates should be interpreted as descriptive/exploratory rather than definitive, as correlation and concordance coefficients are highly sensitive to individual data points at this sample size. Still, principal concordance between these independent methods has been confirmed.

**Fig. 6:**
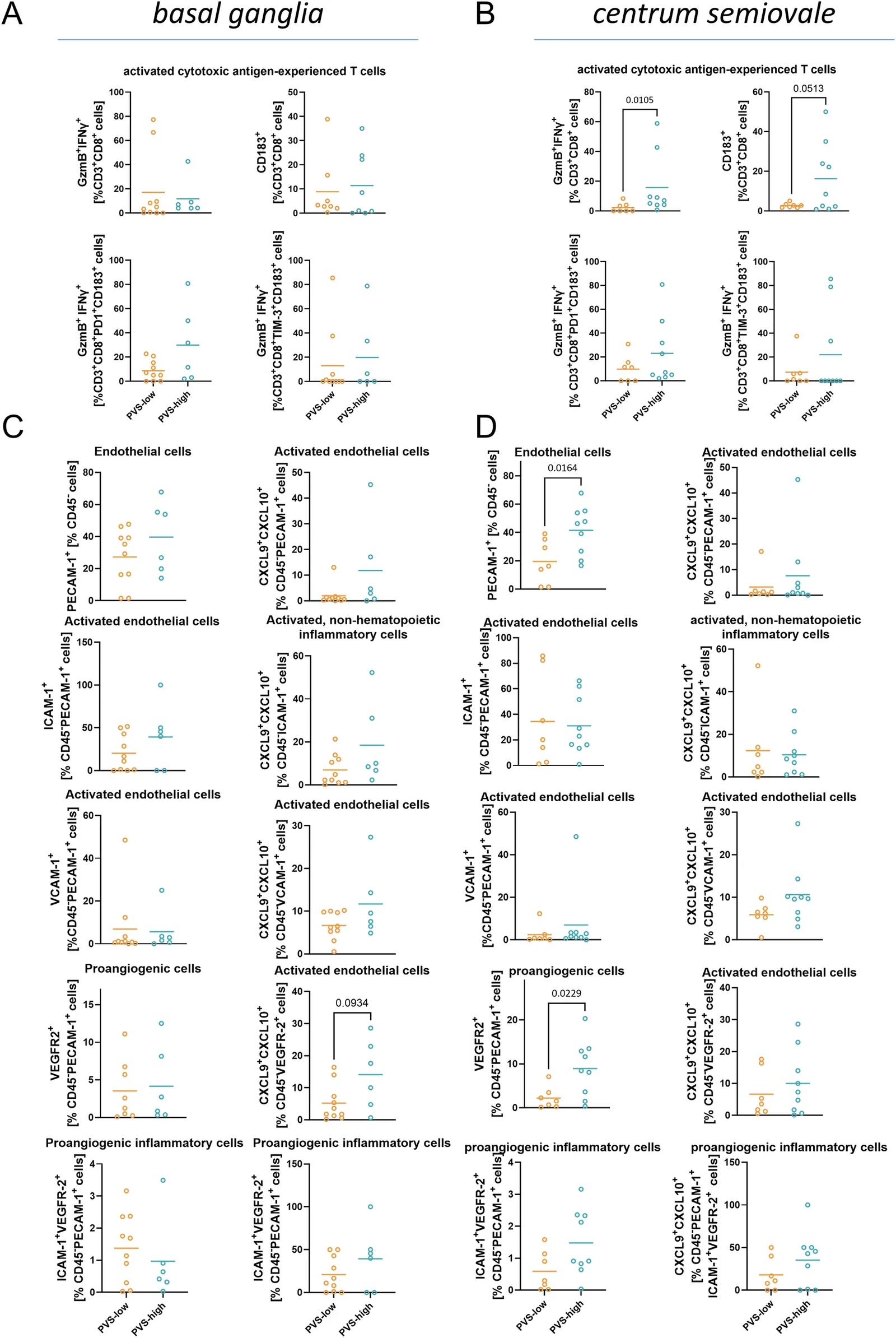
Flow cytometry of BM samples stratified by PVS grade. CD45^+^ and CD45^-^ cell fractions were examined for subtype-specific cell infiltration. Infiltration patterns were stratified by MRI-T2 weighted PVS grade in two distinct anatomic locations, i.e. (**A**, **C**) BG and (**B**, **D**) CSO. For CD45+ fractions, cytotoxicity and exhaustion-related markers were considered (CD3, CD8, PD1, CD183/CXCR3, Granzyme B, and IFNγ). In the CD45- fractions, markers associated with vascular and endothelial cells, involved in T cell trafficking (CD31/PECAM-1, CD54/ICAM-1, CXCL9/CXCL10, CD106/VCAM-1, and CD309/VEGFR2) were analyzed. Cell quantification was done by including the number of viable immune cells, positive for either marker. Given is the % of positive cells after gating of the viable immune cells with additional hierarchical sub-gating to distinguish cellular compartments and activation states. *N* = 16, Unpaired two-tailed Student’s *t* test or Mann–Whitney U test, * p < 0.05.

Additional analysis were performed on CD45^+^ and CD45^-^ subpopulation, to further analyzed the association between PVS and local inflammatory niches (Fig. 6 and supplementary Figs. 6 and 7). Proportions of Granzyme B^+^/IFNγ^+^ cytotoxic T cells were increased in both the BG and CSO PVS^high^ groups and reached statistical significance upon CSO-based stratification (*p* < 0.05, Fig. 6A, B). CD8^+^ cytotoxic T cells additionally showed elevated expression of the migration marker CXCR3, consistent with effector polarization. Although these cells co-expressed PD-1 and TIM-3—markers commonly associated with exhaustion—Granzyme B^+^/IFNγ^+^ levels remained higher in PVS^high^ than in PVS^low^ cases. Together, these findings indicate that PVS^high^ indicates a functionally competent cytotoxic T-cell compartment, in which effector activity is preserved despite the concurrent expression of inhibitory checkpoint molecules.

In the CD45^-^ compartment, we analyzed the association between enhanced infiltration of activated T cells and T cell homing along proangiogenic, inflammatory routes. Stratification by BG revealed minor differences between high and low subgroups, including a modest increase in PECAM-1^+^ endothelial cells with upregulation of ICAM-1 and VCAM-1 (Fig. 6C). In the CSO, PVS^high^ cases were associated with s significantly higher abundance of activated PECAM-1^+^ endothelial cells, co-expressing VCAM-1, CXCL9 and CXCL10 (Fig. 6D). Notably, numbers of VEGFR-2-expressing cells were significantly elevated in the PVS^high^ compared with the PVS^low^ subgroup, with the majority displaying an activated proinflammatory phenotype (ICAM-1^+^VEGFR-2^+^PECAM-1^+^, Fig. 6D). Together, these findings indicate that PVS^high^ is characterized by an activated, chemokine-secreting endothelium that supports directed T-cell recruitment through proangiogenic and inflammatory vascular routes.

**Fig. 7:**
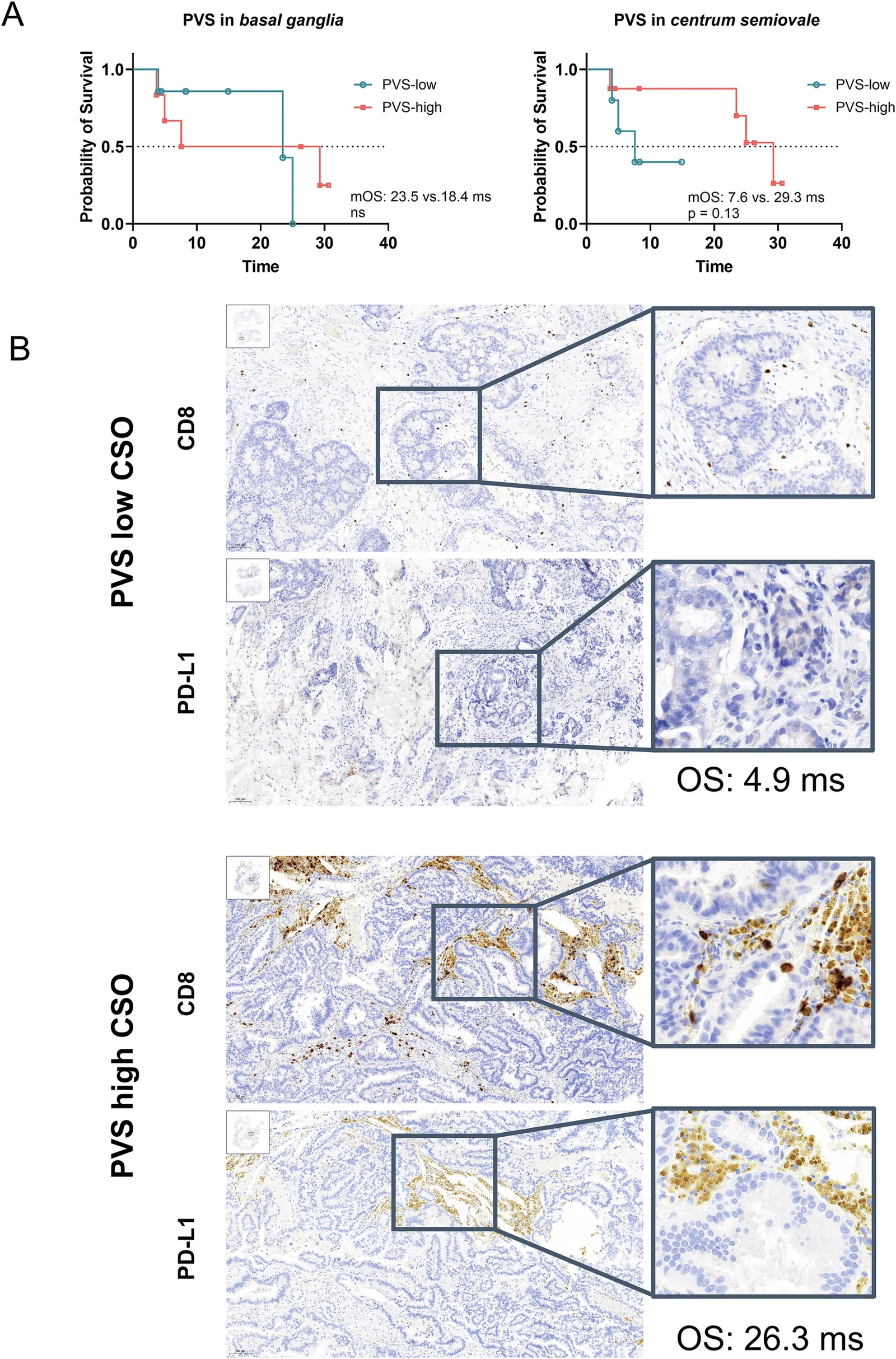
Impact of PVS on overall survival and infiltration in patients receiving immunotherapy. **A** Kaplan-Meier survival curves. Overall survival of patients with BMs stratified by PVS grade. *N* = 13. PVS were rated in two distinct anatomic regions: the basal ganglia (BG) and the centrum semiovale (CSO) underneath the cerebral cortex. For further stratification, PVS were dichotomized into PVS-high and PVS-low. Log rank analysis; mOS—median overall survival; PVS—perivascular spaces. **B** Representative immunohistochemistry of infiltrating CD8^+^ cells and PD-L1 positivity stratified by PVS grade (low vs. high) graded in the centrum semiovale region. The overall survival is additionally given. Original magnification 10×. Scale bar: 100 µM.

Finally, we evaluated whether PVS grade eventually predicts response to immune checkpoint inhibition (Fig. 7). Data from 13 patients treated with anti-PD-1/PD-L1 or CTLA-4 antibodies were available for analysis. Although limited by the small sample size, patients in the PVS-high group (CSO region) showed a trend toward improved survival compared to the PVS-low group (median OS: 29.3 vs. 7.6 months; Fig. 7A). PVS grade also correlated with immune infiltration, with PVS-high tumors exhibiting increased CD8^+^ T-cell density and PD-L1 positivity (Fig. 7B). Together, these exploratory findings indicate that T-cell infiltration along with immune-checkpoint expression correlates with PVS grading in BM patients.

## Discussion

Here, we present evidence for the prognostic relevance of PVS in BM patients, proposing PVS as a potential surrogate marker for tumor immune cell infiltration. This finding holds particular clinical relevance as BM patients, despite advances in immunotherapy, continue to face the poorest prognosis among oncological patients. The absence of reliable predictive biomarkers for patient stratification, coupled with the inherent heterogeneity of treatment responses, further exacerbates this challenge.

By integrating protein-based multiparametric analyses alongside spatial tissue characterization, we have gained critical insights into the immune cell composition of the most abundant immune populations in BMs samples derived from distinct extracranial primary tumors. Clinically, approximately 50% of BM cases are secondary to primary lung cancer^18^, a distribution mirrored in our study cohort. However, the specific immune infiltration pattern in BMs can vary, depending on the primary tumor site^1^. While the limited sample size in our study precluded a tumor-specific analysis, we observed that patient-individual differences rather than distinct anatomic sites of the primary tumors likely contributed to the intertumoral heterogeneity observed.

A recent study by Klemm et al., highlighted the complex microenvironment of BM, where T cells and myeloid cells appear to engage in direct competition, underscoring the need to further dissect these interactions in the context of BM^1^. Another study demonstrated that the brain metastatic niches are either proliferative or inflammatory, shaped by tumor-immune cell interactions^19^. Consistent with this, our study identified a high number of infiltrating immune cells from both the adaptive and innate immune system, with myeloid cells notably outcompeting T cells. We also observed substantial patient-to-patient heterogeneity in immune cell infiltration, which reflects the variability in BM microenvironmental profiles that correlate with patient prognosis. In particular, cytotoxic T cells, whose anti-tumor role is well established, were prognostically relevant, with increased infiltration indicating a favorable outcome. Despite this likely enhanced T-cell-mediated immune response, we additionally found significantly increased expression of the immune checkpoint PD-L1 in survivors, suggesting T cell exhaustion following antigen priming in the draining lymph nodes from extracranial tumors^20^. This paradoxical association has previously been reported in lung cancer BM, where PD-L1 expression, although linked to T cell dysfunction, was nonetheless associated with better prognosis^21^.

Exhausted or chronically activated T cells can persist long-term within the tumor microenvironment, maintaining antigen specificity. Although their direct cytotoxic capacity is often diminished, they may still exert antitumor effects indirectly through sustained cytokine production and modulation of other immune cell subsets, thereby fostering a coordinated antitumoral immune response. Importantly, therapeutic immune-checkpoint inhibition has been shown to further augment T cell infiltration into BM, with infiltrating T cells displaying tumor-specific phenotypes and pronounced activation/exhaustion profiles^22^. Furthermore, we observed a similar positive prognostic relationship with the M1/M2 macrophage ratio, consistent with previous reports that a high abundance of M1 macrophages reflects a proinflammatory microenvironment conducive to improved tumor control^23^. This underscores the complex interplay of immune cell dynamics in BM, which is further shaped by the composition and architecture of the specialized brain microenvironment, including neurons, vascular cells and neuroglial cells, collectively forming the perivascular unit^24,25^. Expanding insights into these neuro–cancer interactions provide a promising framework for the development of neuroscience-guided cancer therapies.

Through in-depth spectral flow cytometry, we here gained a more nuanced understanding of the immune landscape, confirming that infiltrating T cells—predominantly PD1^+^—are immunological markers of prognosis. Our analysis revealed Fas as a key immune engager, likely playing a role in the elimination of tumor target cells by antigen-specific activated cytotoxic T cells. Moreover, our study highlighted the coordinated role of the tumor microenvironment in neutrophil recruitment and functional reprogramming, which may have downstream effects on T cell activity and the overall immune composition in BM^1^.

Another notable finding was the distinct pattern of intratumoral vascularization, linking microvascular stimulation to improved survival. Similar observations have been reported in gliomas, where CD34-positive microvascularity holds prognostic value and vascular remodeling toward hypoxia serves as an early marker of recurrence^26,27^. Hypoxic niches then attract, sequester, and reprogram tumor-associated macrophages and cytotoxic T cells through CCL8 and IL-1β, driving them toward an immunosuppressive phenotype^28^. Our study extends these findings and demonstrates, for the first time, that such microvascular changes are associated with PVS grades. Elevated vascularization indicates an imbalance between stimulatory and inhibitory factors, resulting in a pro-angiogenic state that augments tumor perfusion while permitting immune cell infiltration^29^. Accordingly, we interpret the higher vascularization observed in patients with MRI-detectable PVS as a potential entry point for peripheral T cells into BMs. These T cells—antigen-specific and activated—may in turn exert selective pressure on tumor cells, leading to PD-L1 upregulation, consistent with our observation of higher PD-L1 expression in patients with high PVS. The parallel enrichment of effector-polarized CD8^+^ T cells and proinflammatory, VEGFR-2–expressing endothelial cells suggests a coordinated vascular-immune axis that supports directed T-cell recruitment and sustained effector activity despite concurrent checkpoint engagement. Hence, PVS enlargement is associated with an activated, chemokine-expressing endothelium permissive to effector T-cell homing. Prior studies have also shown that endothelial cells in the PVS provide a specific niche for the macrophage polarization into M2-like phenotypes^30^. This process shapes the microenvironment of BM, leading to the formation of “perivascular cuffs”, a structure which confines immune cells in close proximity to blood vessels, histologically presenting as immune exclusive microenvironment^22^. Our findings of a trend to a more favorable M1/M2 ratio as correlate of PVS grading support these data, positioning macrophages, in addition of cytotoxic T cells, as central surrogate of PVS in BM patients.

Although PVS are often regarded as reflective of glymphatic function, their size may also be influenced by vascular, inflammatory, and structural factors independent of glymphatic activity^31^.

PVS can, for instance, be influenced by chemotherapy where shrinkage is often observed, preceded by alterations in choroid plexus volume, suggesting involvement of the glymphatic system in treatment-related brain changes^32^. As such, evidence on the relationship between PVS size, glymphatic dysfunction, and brain tumors remains conflicting: one study reported no association in BM, whereas another demonstrated impaired glymphatic function and disrupted brain homeostasis in glioma patients^9,33^. These discrepancies underscore the need to consider additional pathophysiological mechanisms when evaluating the role of PVS and glymphatic function in tumor–immune interactions. A recent study even linked PVS in the BG region to cancer-related stroke in patients undergoing radiotherapy^34^, and isolated case reports have described PVS appearing years after brain irradiation for low-grade tumors^35,36^. Notably, these observations have been confined to the BG, whereas the CSO region—identified in our study as a prognostic immune gatekeeper and surrogate marker of T cell infiltration—has not been analyzed.

These region-specific differences in clinical associations may reflect fundamental anatomical and functional heterogeneity between the CSO and BG. The CSO, as part of the deep white matter, is characterized by distinct interstitial fluid dynamics and perivascular drainage pathways, which may facilitate immune cell trafficking and antigen exchange. In contrast, the BG exhibits a different vascular architecture and metabolic profile, which may influence perivascular flow and immune accessibility. Such differences in vascular organization and local immune microenvironment may underlie the divergent prognostic relevance of PVS observed in our study, although mechanistic validation is required. Accordingly, future studies should focus on integrating high-resolution vascular imaging and spatial immune profiling.

Given that PVS differ in prevalence and extend between regions and that their associations with vascular risk factors and inflammatory markers can vary^4^, it is essential to evaluate PVS in both anatomical compartments, particularly within the immune contexture of brain metastases, which remains understudied. PVS provide the anatomical and fluidic framework that interfaces with perivascular niches, facilitating the exchange of cerebrospinal fluid, metabolites, and immune mediators, thereby shaping cellular dynamics involved in immune surveillance, stem cell maintenance, and tumor–stroma interactions^37,38^. Hence, PVS and associated niches have been described as protective microenvironments that shield (cancer) stem cells from environmental insults^39^, including chemo- and radiotherapy. Importantly, our data indicate that these niches along the PVS can also serve as sites for antigen priming of T cells, thereby exhibiting functional features reminiscent of lymph nodes.

From a translational perspective, PVS grading may have potential as a non-invasive imaging biomarker that complements established prognostic frameworks. In particular, integration of PVS grading with widely used clinical scoring systems, such as GPA, could enhance risk stratification by incorporating imaging-derived information that may reflect the tumor immune microenvironment. Given the observed association between higher PVS grade and increased immune cell infiltration, PVS may provide incremental value in identifying biologically distinct patient subgroups beyond conventional clinical parameters. However, several considerations must be addressed prior to clinical implementation. These include the need for standardized imaging acquisition and grading criteria, assessment of inter-observer reproducibility, and validation of its additive prognostic value in large, prospective, and ideally multicenter cohorts. Moreover, the extent to which PVS is influenced by confounding factors, such as treatment effects or comorbid vascular changes, remains to be clarified in detail. Therefore, while our findings support the potential clinical utility of PVS, further studies are required to establish its robustness, generalizability, and integration into routine prognostic models.

Finally, this study has several limitations. A key limitation of this study is the relatively small cohort, which may limit statistical power. While inclusion of patients from two centers enhances generalizability compared to a single-center design, the sample remains constrained, particularly for analyses requiring complete multimodal datasets (PVS imaging and immune profiling). In addition, the cohort does not fully capture the spectrum of cancer-associated BMs, with melanoma- and breast cancer–derived cases underrepresented. Accordingly, subgroup analyses—especially those related to immunotherapy response—should be interpreted as exploratory. Larger, ideally multicenter studies with independent validation cohorts are needed to confirm the robustness and generalizability of these findings.

In addition, residual intratumoral spatial heterogeneity remains an important limitation. Future studies incorporating spatially resolved imaging approaches to delineate immune “hot” and “cold” niches within tumor samples will be essential to further refine the patients individual BM microenvironments. Another limitation is the use of two different methods—immunohistochemistry and flow cytometry—to correlate PVS grading with tumor immune infiltration. While this dual approach provided valuable insights, it may introduce technical variability and limit direct comparability, particularly in relation to spatial intratumoral heterogeneity and vascularity. Moreover, flow cytometry cannot fully capture the complexity of tumor–stromal, and immune interactions, and immunohistochemistry is restricted in the number of immune populations it can resolve, precluding assessment of co-expression patterns such as activation and exhaustion markers in specific immune cell subtypes. Nevertheless, the combined approaches provide sufficient information to map PVS findings with immune features.

In summary, to our knowledge, this is the first report demonstrating an association between PVS, immune infiltration, and patient prognosis. We show that PVS constitute critical hubs for immune cells infiltrating BMs, likely contributing to antitumor immune responses. Given their characteristic imaging and spatial patterns, sophisticated neuroimaging techniques may allow non-invasive evaluation of PVS as indicators of tumor-related metabolic stress and immune dysregulation. Prospective integration of PVS imaging with tumor biology and treatment response has the potential to guide therapeutic strategies and ultimately improve patient outcomes.

## Supplementary information

Supplementary Information

Supplementary Table 1

Supplementary Table 2

## Data Availability

All relevant data are available from the authors upon request to claudia.maletzki@med.uni-rostock.de as CM will be responsible for replying to this request.
