# Supplementary material for: Perivascular spaces are associated with T cell trafficking and may have prognostic relevance in brain metastases
Source: Commun Med (Lond). 2026 Oct 19;6:542. doi: 10.1038/s43856-026-01918-7 (PMC13680621; doi:10.1038/s43856-026-01918-7)
Supplement: Supplementary file 1 [file 43856_2026_1918_MOESM1_ESM.pdf]

Supplementary Figure 1: Representative images of T2-weighted MRI scans from BM patients. PVS measurement was done using T2-weighted axial or coronal MRI sequences. PVS were rated in two distinct anatomic regions: the basal ganglia (BG) and the centrum semiovale (CSO). Severity was determined based on individual PVS counts, with each region graded separately as follows: grade I (<10 PVS), grade II (10–20), grade III (20–40), and grade IV (>40).

Supplementary Figure 2: Outcome of patients with brain metastases (BM) stratified by GPA score. (**A, B**) Kaplan Meier survival curves showing overall survival of patients with BMs. Primary extracranial tumor location was in the lung. N = 26. PVS were rated in two distinct anatomic regions: the basal ganglia (BG) and the centrum semiovale (CSO). PVS were dichotomized into PVS-high and PVS-low. Log rank analysis; $p < 0.05$ *vs.* PVS-low. mOS – median overall survival; PVS – perivascular spaces.

Supplementary Figure 3: Immunohistochemistry of BM samples. Representative images for each analyzed immune and vascular marker are shown, with each showing either low or high infiltration. Original magnification 10x. Scale bar: 100 µm.

Supplementary Figure 4: Immunohistochemistry of BM samples. Quantitative analysis of infiltrating immune cells on whole tumor sections. Immune cell infiltration patterns ($CD8^+$ T cells and M1 or M2-like macrophages) were assessed across whole-slide images, stratified by either total or intratumoral infiltration. No significant differences were detected within single regions.

Supplementary Figure 5: Concordance between IHC and flow cytometry (FC) measurements was assessed using Pearson's correlation coefficient (r), Spearman's rank correlation coefficient (ρ), and Lin's concordance correlation coefficient (CCC). Given the small sample size (n = 5 per marker), these concordance estimates should be

interpreted as descriptive/exploratory rather than definitive, as correlation and concordance coefficients are highly sensitive to individual data points at this sample size. Still, large consistency could be confirmed, even in this small data set.

Supplementary Figure 6: Gating strategy for $CD45^{+}$ cell fractions isolated from whole tumor tissues. Hierarchical gating was performed on viable cells as shown in the figure.

Supplementary Figure 7: Gating strategy for $CD45^{-}$ cell fractions isolated from whole tumor tissues. Hierarchical gating was performed on viable cells as shown in the figure.

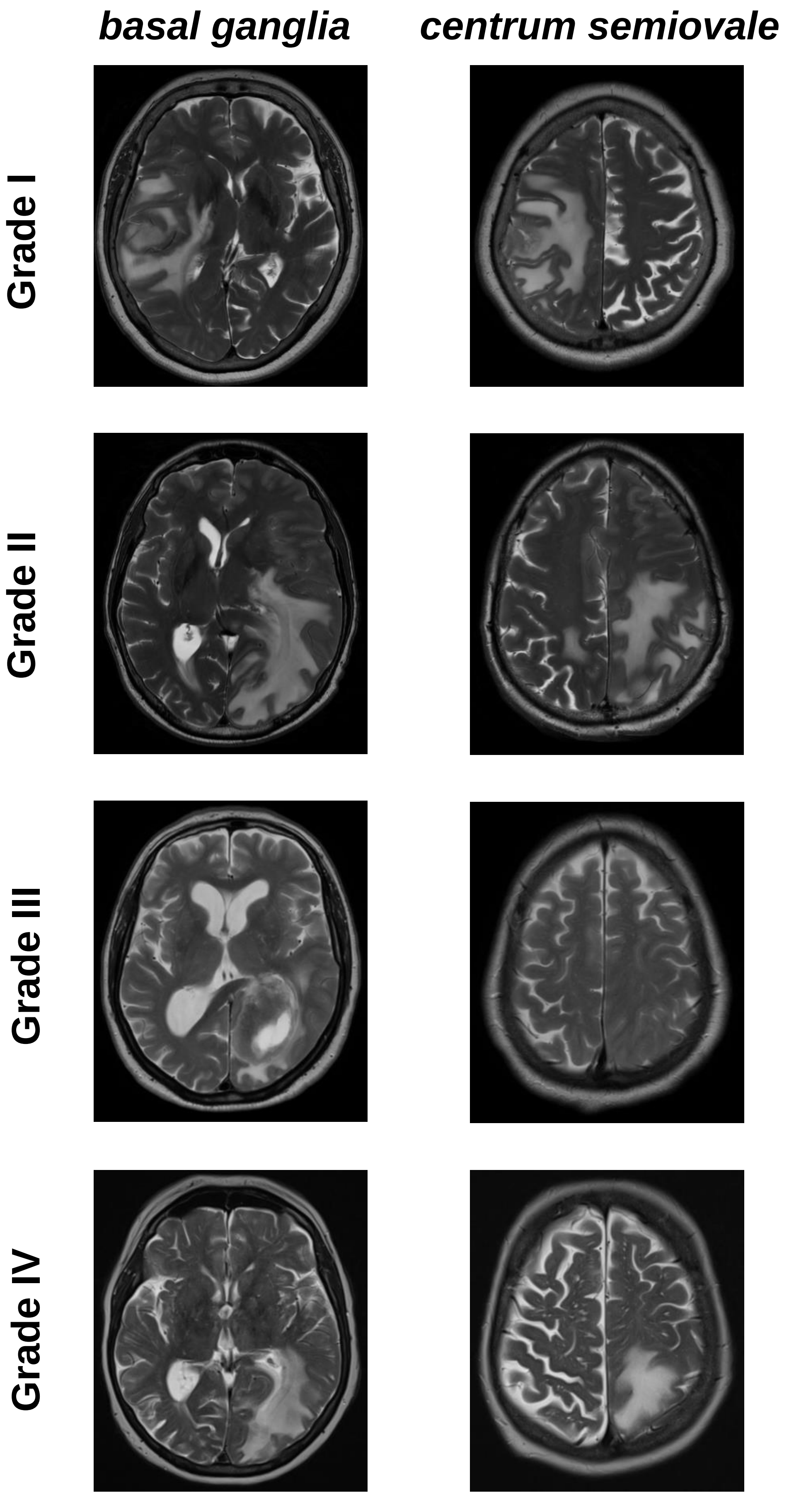

Supplementary Figure 1

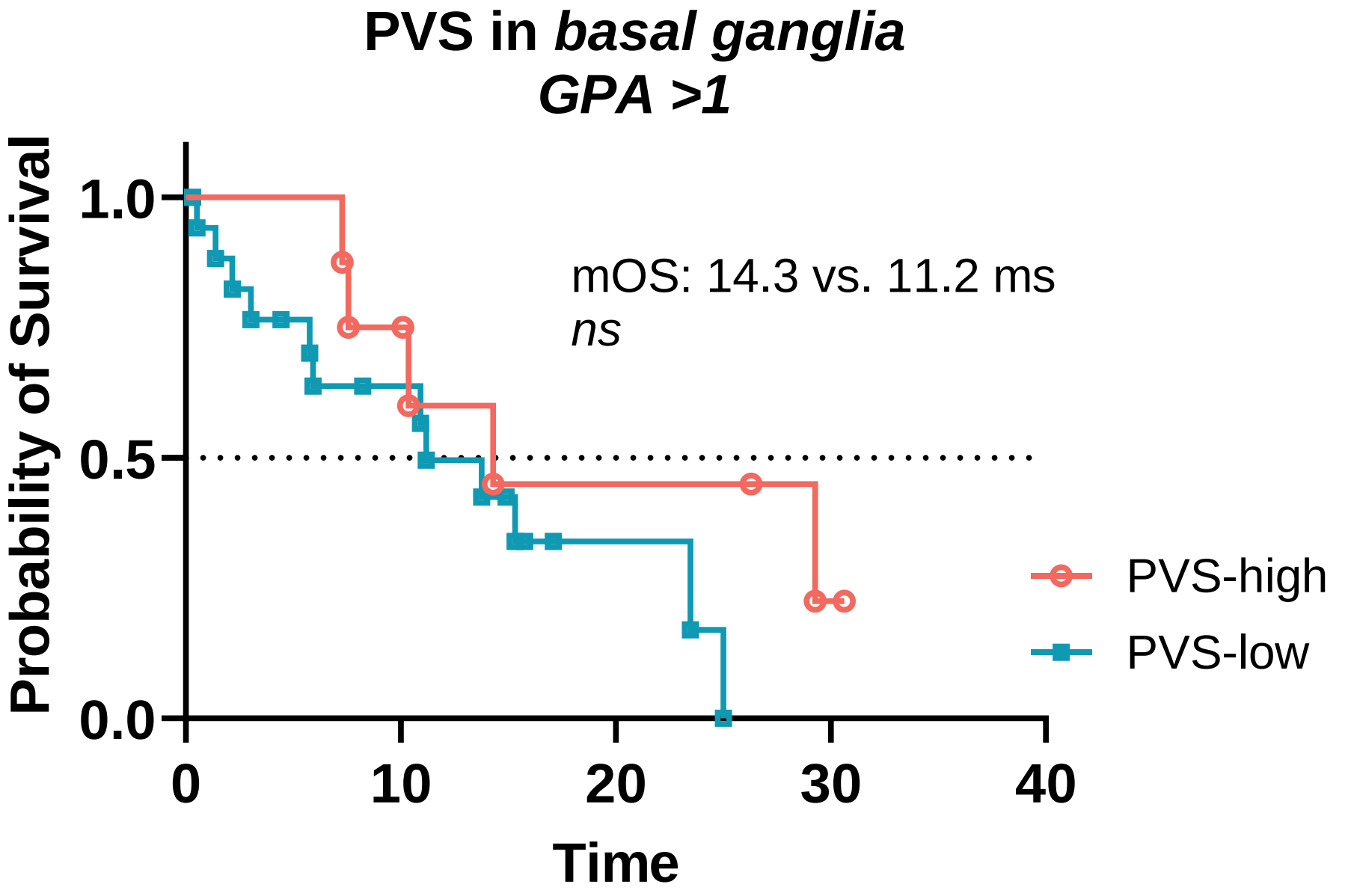

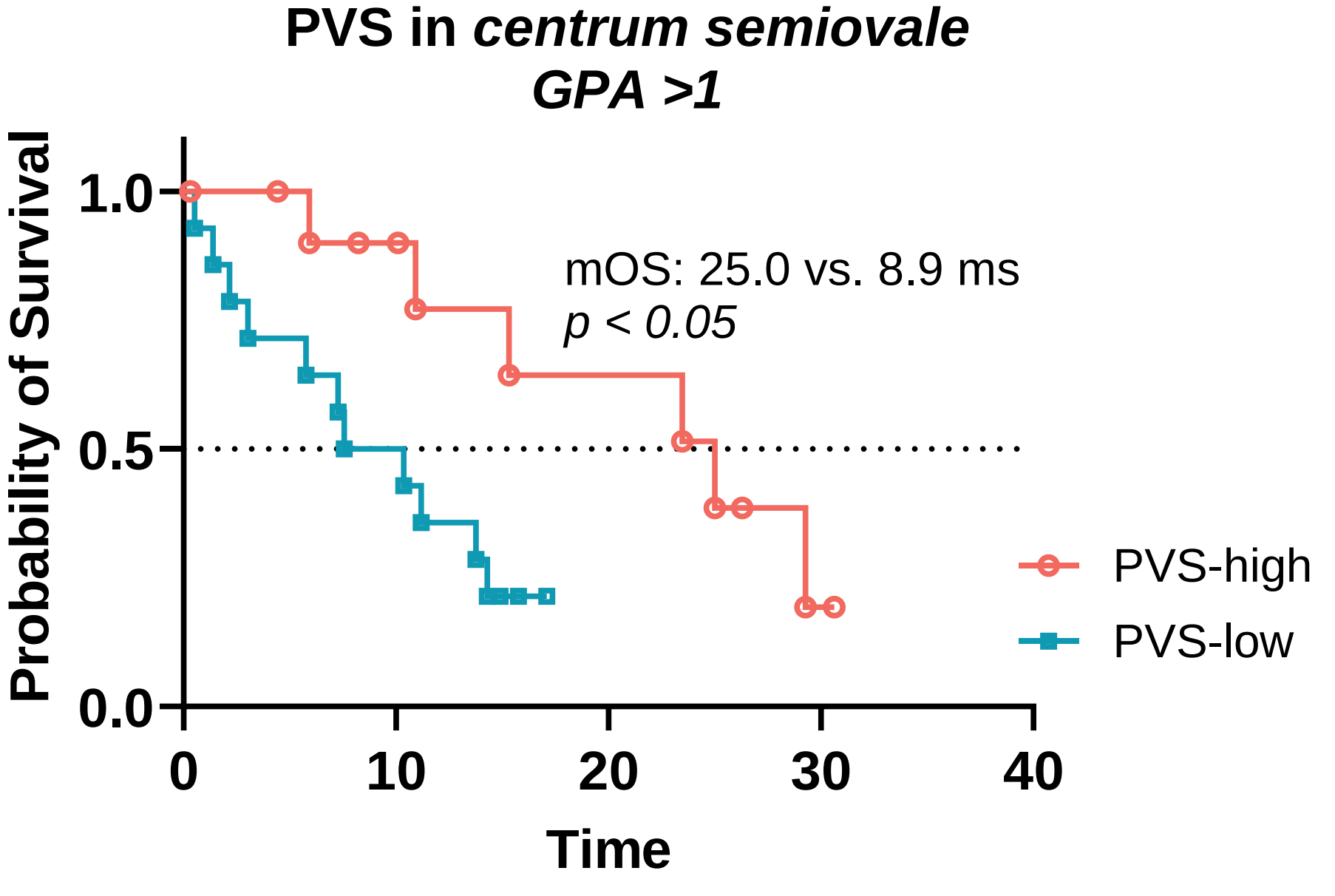

Hazard Ratio (logrank)	A/B
Ratio (and its reciprocal)	0.3491
95% CI of ratio	0.1317 to 0.9250

Supplementary Figure 2

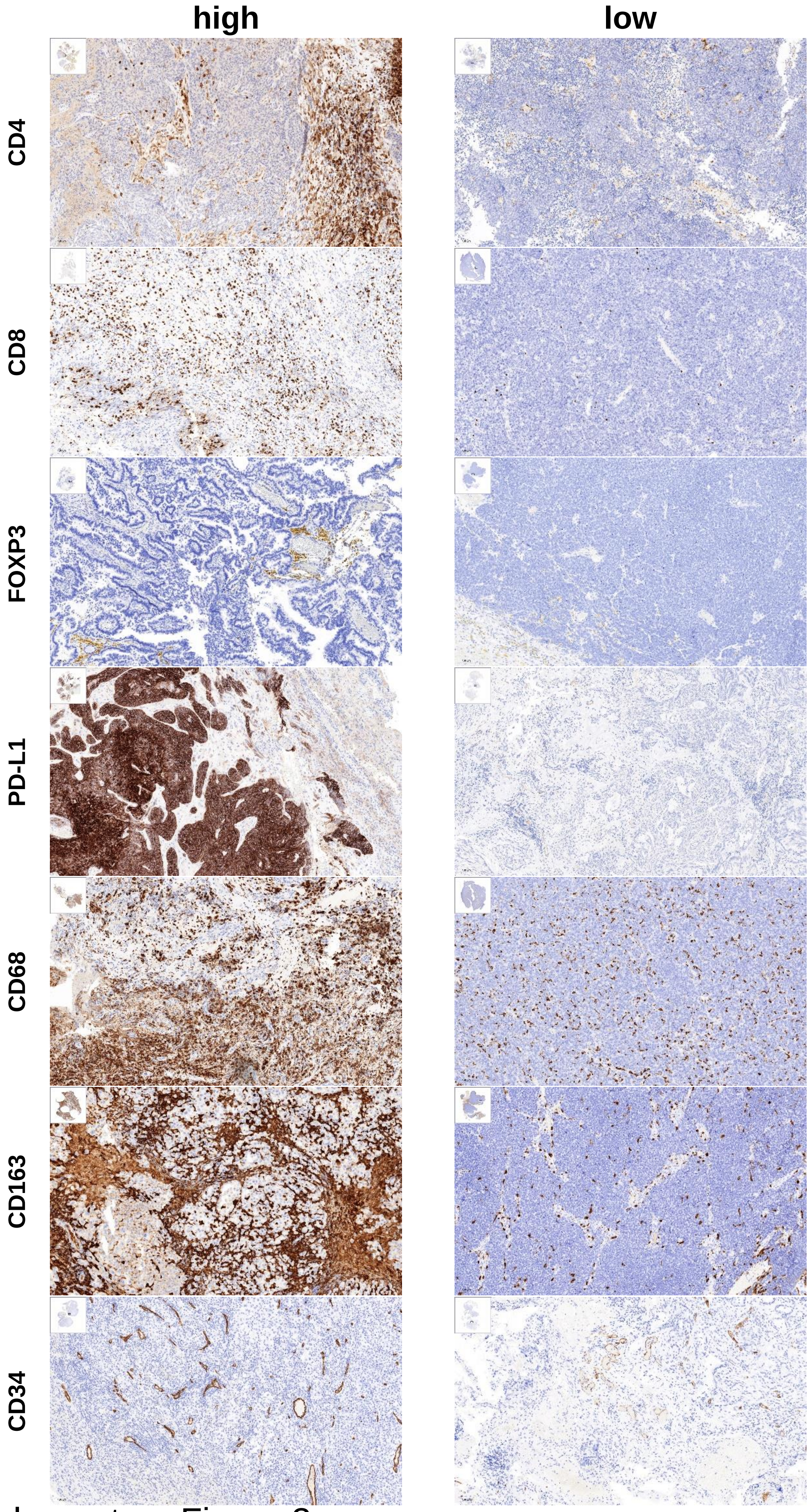

Supplementary Figure 3

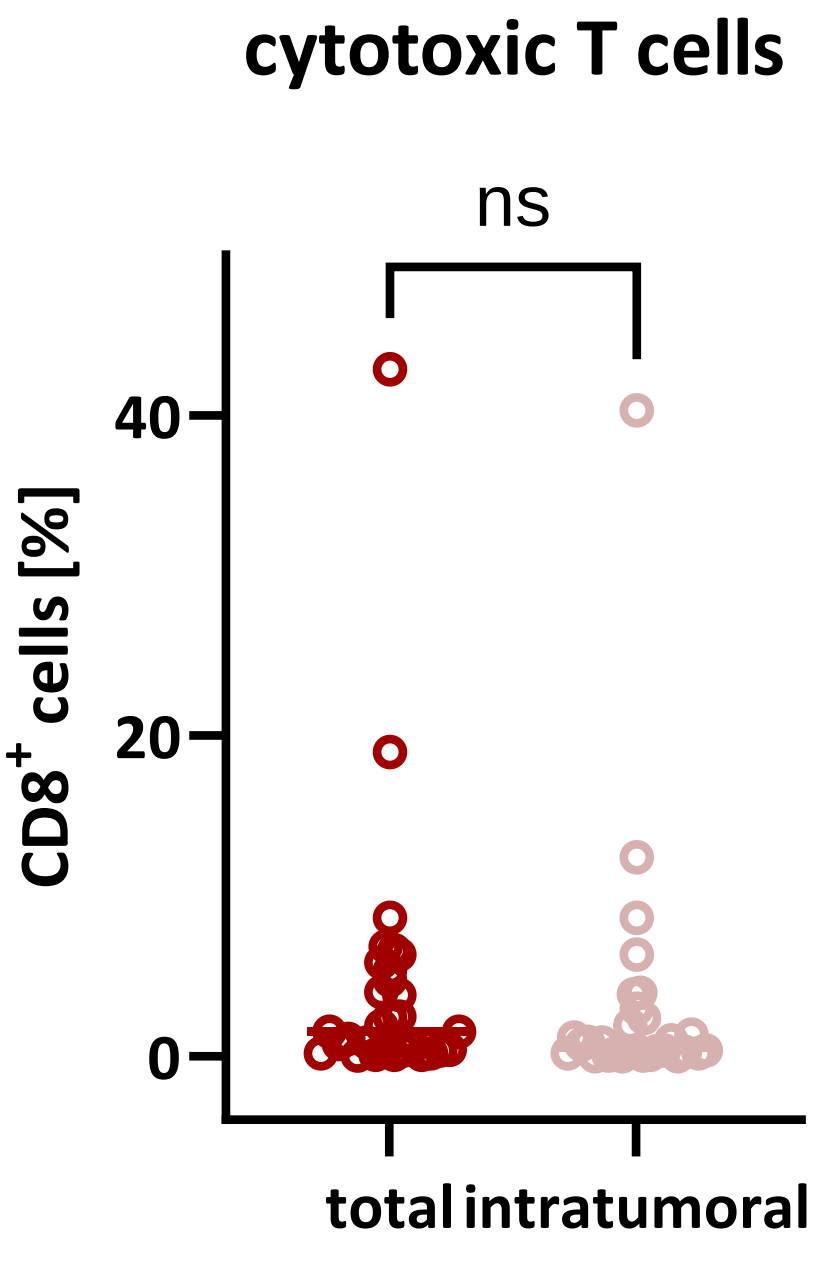

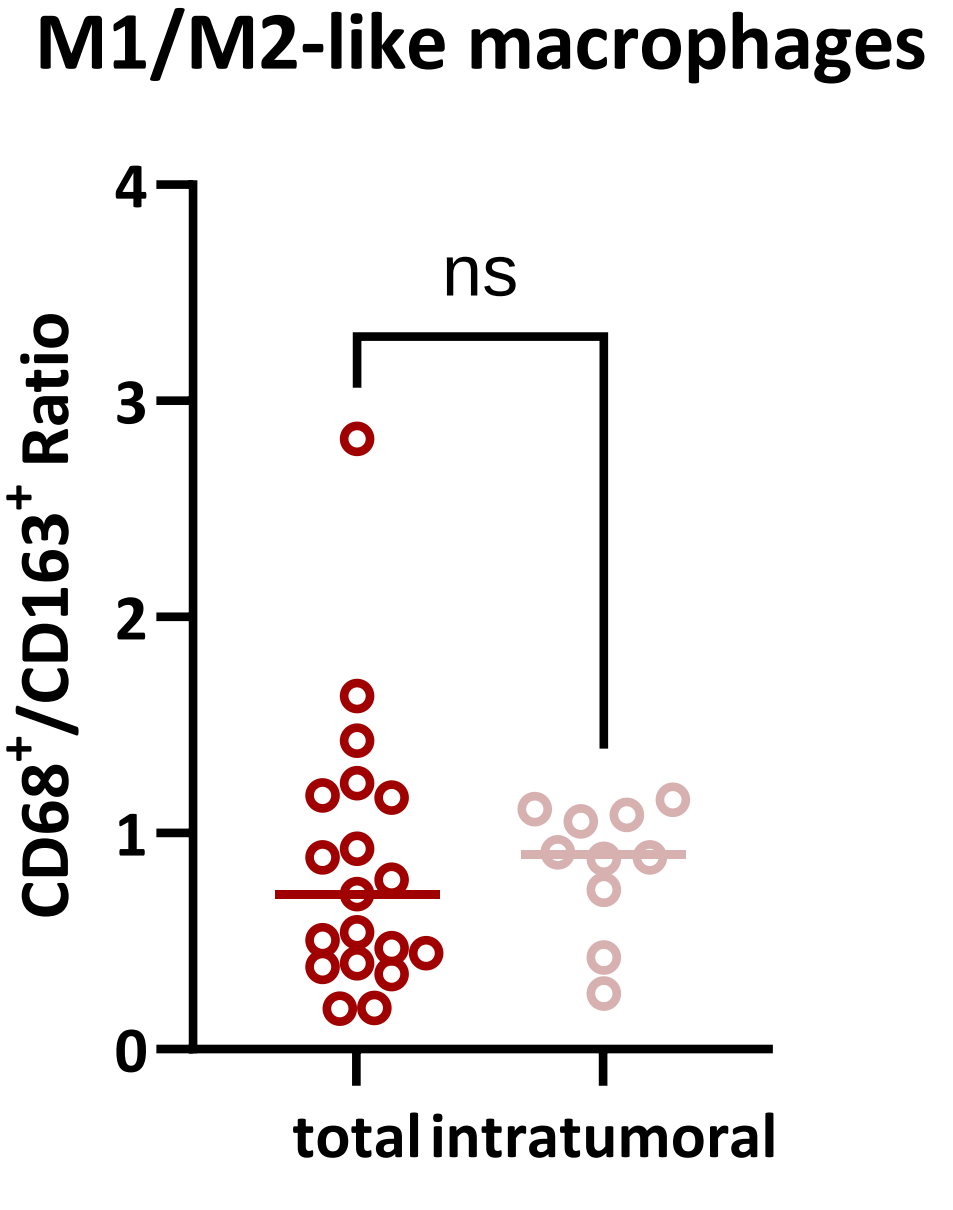

Supplementary Figure 4

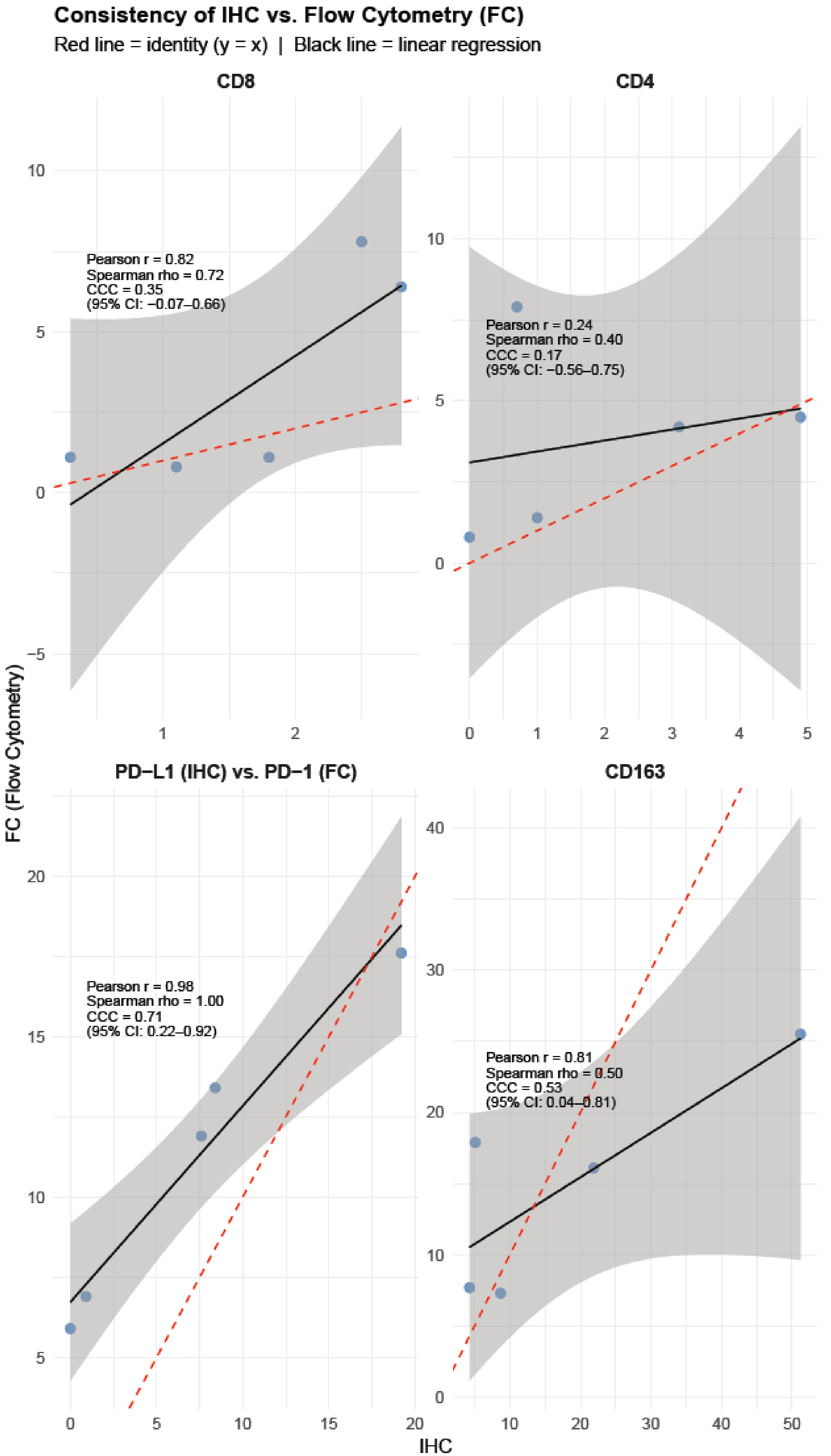

Supplementary Figure 5

$CD45^+$ Fraction

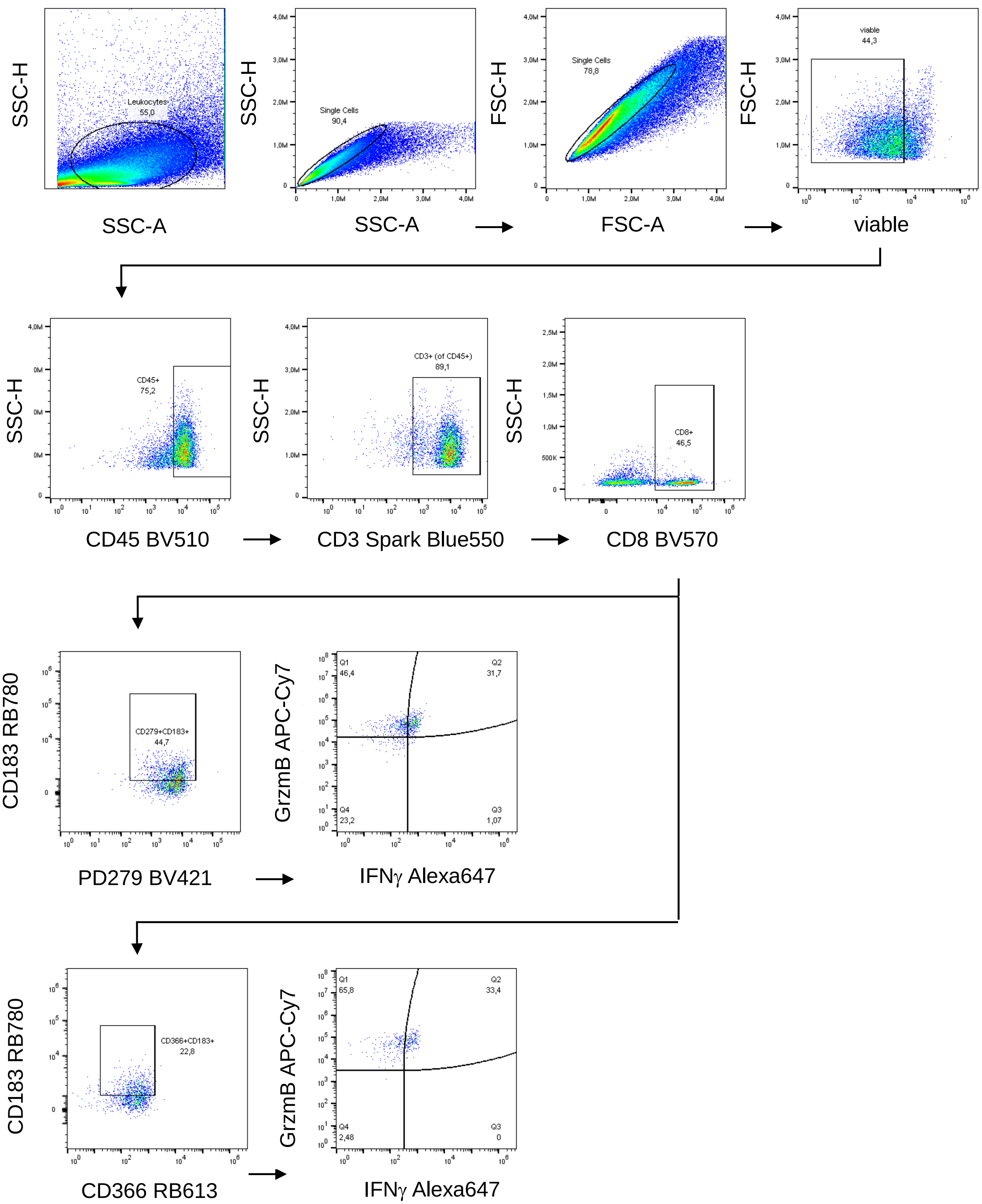

Supplementary Figure 6

CD45- Fraction

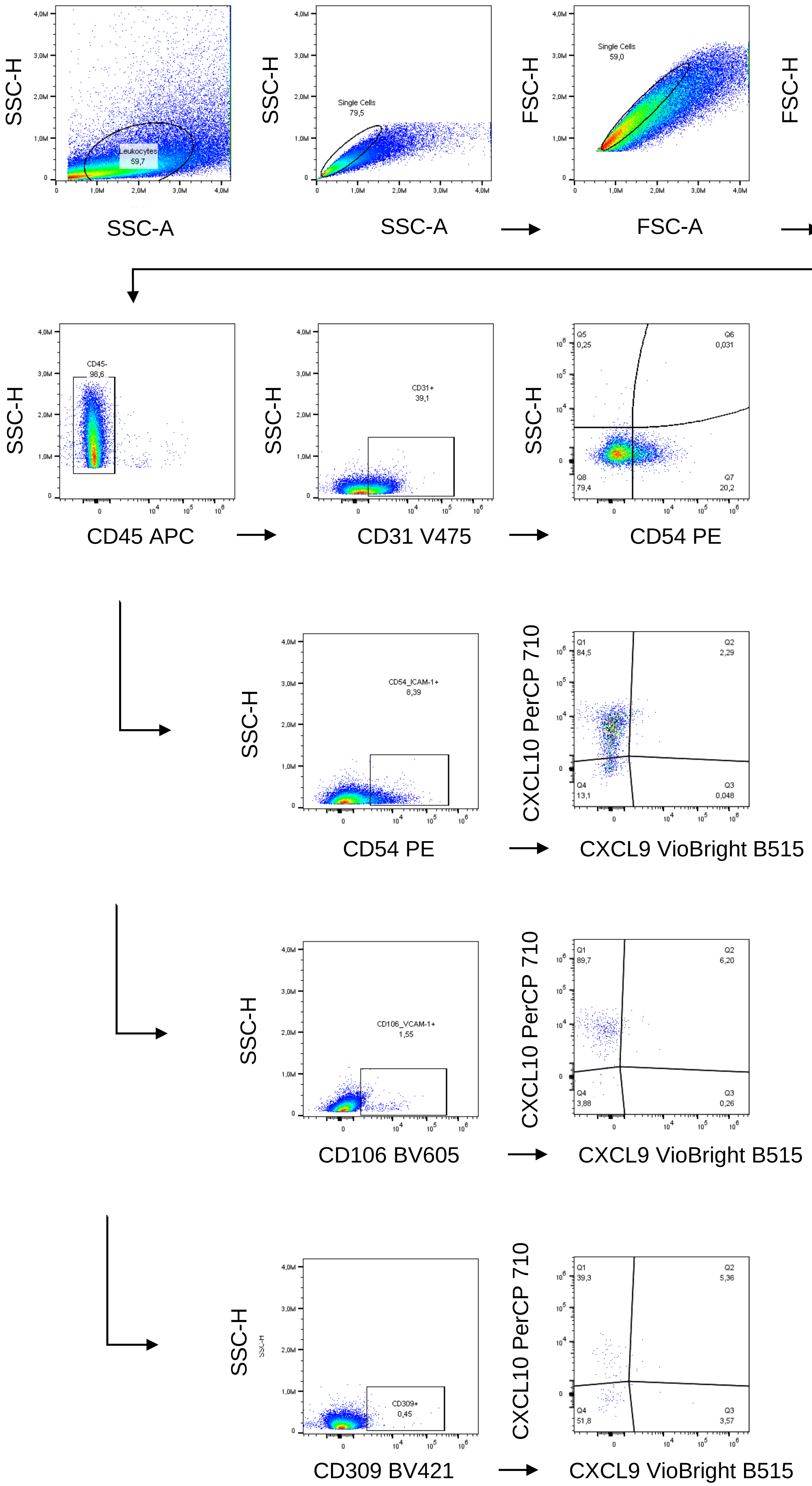

Supplementary Figure 7
