# Supplementary material for: Perivascular spaces are associated with T cell trafficking and may have prognostic relevance in brain metastases
Source: Commun Med (Lond). 2026 Oct 19;6:542. doi: 10.1038/s43856-026-01918-7 (PMC13680621; doi:10.1038/s43856-026-01918-7)
Supplement: Supplementary file 2 [file 43856_2026_1918_MOESM2_ESM.docx]

**Supplementary Table 1: Multiple comparison adjustment using the two-stage linear step-up procedure of Benjamini, Krieger, and Yekutieli.**

|  | Mean difference | Discovery?* | q value Individual | P Value |
| --- | --- | --- | --- | --- |
| CD3^+^CD4^+^ vs. CD3^+^CD8^+^ | 0.3042 | No | 0.4537 | 0.6481 |
| CD3^+^CD4^+^ vs. PD-1^+^ | -10.13 | Yes | 0.0034 | **0.0003** |
| CD3^+^CD4^+^ vs. CD8^+^PD-1^+^ | -15.53 | Yes | 0.0059 | **0.0023** |
| CD3^+^CD4^+^ vs. CD14+CD16+ | -0.1931 | No | 0.5690 | 0.9483 |
| CD3^+^CD4^+^ vs. CD4^+^CD95^+^ | -3.722 | No | 0.0902 | 0.0787 |
| CD3^+^CD4^+^ vs. CD8^+^CD95^+^ | -0.6065 | No | 0.4537 | 0.6434 |
| CD3^+^CD8^+^ vs. PD-1^+^ | -10.43 | Yes | 0.0034 | **0.0008** |
| CD3^+^CD8^+^ vs. CD8^+^PD-1^+^ | -15.83 | Yes | 0.0045 | 0.0014 |
| CD3^+^CD8^+^ vs. CD14^+^CD16^+^ | -0.4973 | No | 0.5649 | 0.8765 |
| CD3^+^CD8^+^ vs. CD4^+^CD95+ | -4.026 | No | 0.1102 | 0.1049 |
| CD3^+^CD8^+^ vs. CD8^+^CD95^+^ | -0.9108 | No | 0.3732 | 0.4740 |
| PD-1^+^ vs. CD8^+^PD-1^+^ | -5.513 | No | 0.3183 | 0.3789 |
| PD-1^+^ vs. CD14^+^CD163^+^ | 9.936 | Yes | 0.0191 | 0.0122 |
| PD-1^+^ vs. CD4^+^CD95^+^ | 6.407 | Yes | 0.0073 | **0.0035** |
| PD-1^+^ vs. CD8^+^CD95^+^ | 9.523 | Yes | 0.0034 | **0.0007** |
| CD8^+^PD-1^+^ vs. CD14^+^CD163^+^ | 15.41 | Yes | 0.0199 | **0.0142** |
| CD8^+^PD-1^+^ vs. CD4^+^CD95^+^ | 12.02 | No | 0.0674 | 0.0535 |
| CD8^+^PD-1^+^ vs. CD8^+^CD95^+^ | 14.96 | Yes | 0.0081 | **0.0045** |
| CD14^+^CD163^+^ vs. CD4^+^CD95^+^ | -3.529 | No | 0.2624 | 0.2915 |
| CD14^+^CD163^+^ vs. CD8^+^CD95^+^ | -0.4135 | No | 0.5649 | 0.8966 |
| CD4^+^CD95^+^ vs. CD8^+^CD95^+^ | 3.115 | No | 0.1156 | 0.1192 |

* FDR threshold (1%)
