# Supplementary material for: Perivascular spaces are associated with T cell trafficking and may have prognostic relevance in brain metastases
Source: Commun Med (Lond). 2026 Oct 19;6:542. doi: 10.1038/s43856-026-01918-7 (PMC13680621; doi:10.1038/s43856-026-01918-7)
Supplement: Supplementary file 3 [file 43856_2026_1918_MOESM3_ESM.docx]

| PVS CS  vs. | Mean difference | Discovery?* | q value Individual | P Value |
| --- | --- | --- | --- | --- |
| CD3^+^CD4^+^ | -3.907 | No | 0.1230 | 0.0730 |
| CD3^+^CD8^+^ | -3.539 | No | 0.1584 | 0.1568 |
| PD1^+^ | -15.26 | Yes | 0.0001 | **<0.0001** |
| CD8^+^PD1^+^ | -19.72 | No | 0.0173 | 0.0069 |
| CD14^+^CD163^+^ | -1.506 | No | 0.1802 | 0.2141 |
| PVS BG | 0.2857 | No | 0.1584 | 0.1370 |

**Supplementary Table 2: Multiple comparison adjustment using the two-stage linear step-up procedure of Benjamini, Krieger, and Yekutieli.**

* FDR threshold (1%)
